# Diverse patterns of molecular changes in the mechano-responsiveness of focal adhesions

**DOI:** 10.1038/s41598-018-20252-0

**Published:** 2018-02-01

**Authors:** Rahuman S. Malik-Sheriff, Sarah Imtiaz, Hernán E. Grecco, Eli Zamir

**Affiliations:** 1 Department of Systemic Cell Biology, Max Planck Institute of Molecular Physiology, Dortmund, Germany; 2 Department of Cellular Biophysics, Max Planck Institute for Medical Research, Heidelberg, Germany; 3 Present Address: Department of Physics, FCEN, University of Buenos Aires and IFIBA, CONICET, Buenos Aires, Argentina; 4 Present Address: European Bioinformatics Institute, European Molecular Biology Laboratory, Hinxton, Cambridge, UK

## Abstract

Focal adhesions anchor contractile actin fibers with the extracellular matrix, sense the generated tension and respond to it by changing their morphology and composition. Here we ask how this mechanosensing is enabled at the protein-network level, given the modular assembly and multitasking of focal adhesions. To address this, we applied a sensitive 4-color live cell imaging approach, enabling monitoring patterns of molecular changes in single focal adhesions. Co-imaging zyxin, FAK, vinculin and paxillin revealed heterogeneities in their responses to Rho-associated kinase (ROCK)-mediated perturbations of actomyosin contractility. These responses were rather weakly correlated between the proteins, reflecting diverse compositional changes in different focal adhesions. This diversity is partially attributable to the location of focal adhesions, their area, molecular content and previous contractility perturbations, suggesting that integration of multiple local cues shapes differentially focal adhesion mechano-responsiveness. Importantly, the compositional changes upon ROCK perturbations exhibited distinct paths in different focal adhesions. Moreover, the protein exhibiting the strongest response to ROCK perturbations varied among different focal adhesions. The diversity in response patterns is plausibly enabled by the modular mode of focal adhesions assembly and can provide them the needed flexibility to perform multiple tasks by combining optimally a common set of multifunctional components.

## Introduction

Focal adhesions are multi-molecular structures along the plasma membrane that mediate the anchoring and mechanical coupling of contractile actin stress fibers with the extracellular matrix^1^. This coupling enables cells to pull themselves forward during migration, shape their morphology, sense the mechanical properties of the matrix and re-organize the matrix^2–6^. The mechanical linkage between stress fibers and extracellular matrix in focal adhesions is mediated via receptors of the integrin family and a large number of proteins, collectively termed the integrin adhesome^1,7^. To achieve their functions, focal adhesions sense, and respond to, the mechanical forces applied on them^8,9^. In general, elevation of contractile forces promotes focal adhesions assembly, while inhibition of contractility causes their gradual disassembly^6,9^. At the molecular-level, the mechanosensing of focal adhesions is mediated by components that get an open, active conformation upon mechanical stretching, such as talin and p130Cas^10,11^. The sensing of mechanical forces at the molecular level propagates within the focal adhesion, affecting its molecular content and morphology. Fluorescence recovery after photobleaching experiments showed that inhibition of actomyosin contractility affects differently the binding constants of the various components in focal adhesions^12–14^. This leads to distinct dissociation rates of proteins from focal adhesions upon actomyosin inhibition, in which on average zyxin and VASP exit most rapidly, followed by talin, paxillin, ILK and then FAK, vinculin and kindlin-2^13^. However, it is still unclear what are the principles by which collective molecular changes give rise to the mechanosensing of focal adhesions at the protein-network level.

Two fundamental properties of focal adhesions have to be considered in order to address comprehensively their mechanosensing at the protein-network level. The first property is the modular mode of focal adhesions assembly, arising from the capability of integrin adhesome proteins to interact with each other in alternative manners to form different types of adhesion sites and variable states of these types^1,15–18^. While modular assembly provides the integrin adhesome flexibility in terms of the structures that it can form, it poses an apparent engineering challenge how to ensure a correct assembly solely by self-organization of the components^15^. Hence, it is not clear how focal adhesions can rapidly change in response to force in a correct manner, and how modular assembly could be beneficial for their mechanosensing. The second important feature of focal adhesions is their multitasking, manifested by their capability to sense multiple types of internal and external cues and generate multiple outputs related to adhesion strength, signaling and matrix modulation. Importantly, multitasking is also present at the level of the individual components of focal adhesions, as each of them typically contains multiple domains which are involved, directly or indirectly, in a variety of the focal adhesion functions. In order to illuminate principles underlying the mechanosensing of focal adhesions at the protein-network level, it is important to examine the dynamic patterns of molecular changes occurring in single focal adhesions. However, monitoring such patterns is challenged by the difficulty to co-image quantitatively multiple components located in the same structures in live cells^19^.

In this work we explore the mechanosensing of focal adhesions at the protein-network level. For this purpose we implemented a sensitive approach to co-image the levels of four proteins in single focal adhesions in live cells. We applied this approach for monitoring the levels of paxillin, vinculin, FAK and zyxin in single focal adhesions responding to inhibition and recovery of actomyosin contractility. ROCK promotes actomyosin contractility by phosphorylating myosin light chain and inactivating myosin light chain phosphatase^20^. Thus, acute and reversible perturbations of actomyosin contractility were achieved using the ROCK inhibitor Y-27632^21^. The results show that the responses of paxillin, vinculin, FAK and zyxin to ROCK perturbations in focal adhesions are variable and only moderately correlated between the proteins. Different proteins exhibited different patterns of correlations with the area of the focal adhesions, their molecular content and previous ROCK perturbations, suggesting that they are differentially affected by the various local cues. Strikingly, we found that the combined pattern of changes in the levels of paxillin, vinculin, FAK and zyxin upon ROCK perturbations is considerably different among single focal adhesions. Furthermore, in different focal adhesions different proteins exhibited the strongest response to ROCK perturbations. Hence, this work reveals a role of the modular assembly of focal adhesions in their mechanosensing, as it enables them to shape flexibly their compositional changes in order to assimilate force and additional local cues. In turn, the flexibility in the combined response pattern of the proteins can enable focal adhesions to carry out multiple tasks using a set of proteins, each is contributing to multiple tasks differently.

## Results

### Four-color live cell imaging of adhesion sites

A fundamental barrier in studying cell-matrix adhesion sites arises from their molecular heterogeneity and large number of components^1,7,16^. Coping with this barrier requires the monitoring of multiple components in individual adhesion sites^17,22^. We previously reported 5-color imaging of cell-matrix adhesion sites in fixed cells, achieved by combination of orthogonal immunofluorescence labelings, small molecules and tagging with fluorescent proteins^17^. Along this line, we recently achieved imaging of 10 different components in single focal adhesions using cyclic immunofluorescence imaging^15^ – a techniques in which fixed cells are subjected to serial cycles of immunolabeling, imaging and bleaching. While multiplexed imaging of adhesion sites in fixed cells uncovers their composition diversity, for studying dynamic patterns of molecular changes in focal adhesions it is essential to perform multiplexed imaging in live cells. Although a large palette of fluorescent proteins with different emission and excitation spectra is available^23^, combining them for multicolor imaging is strongly hampered by their spectral overlap^19^. Multispectral imaging and linear unmixing can provide a good solution for cases in which most of the labeled components do not overlap spatially within the cell and that the co-localized ones are present in comparable levels^19,24–26^. However in the case of focal adhesions, the components are localized in the same small structures and could have highly incomparable levels^17,19^. In such cases, spectral imaging and unmixing are sensitive to noise and prone to errors in quantifying the contribution of each fluorescent protein to the integrated measured spectra in each pixel^19^. Therefore, to achieve quantitative multicolor imaging of adhesion sites it is required to use a combination of fluorescent proteins that can be spectrally separated without unmixing.

**Figure 1:**
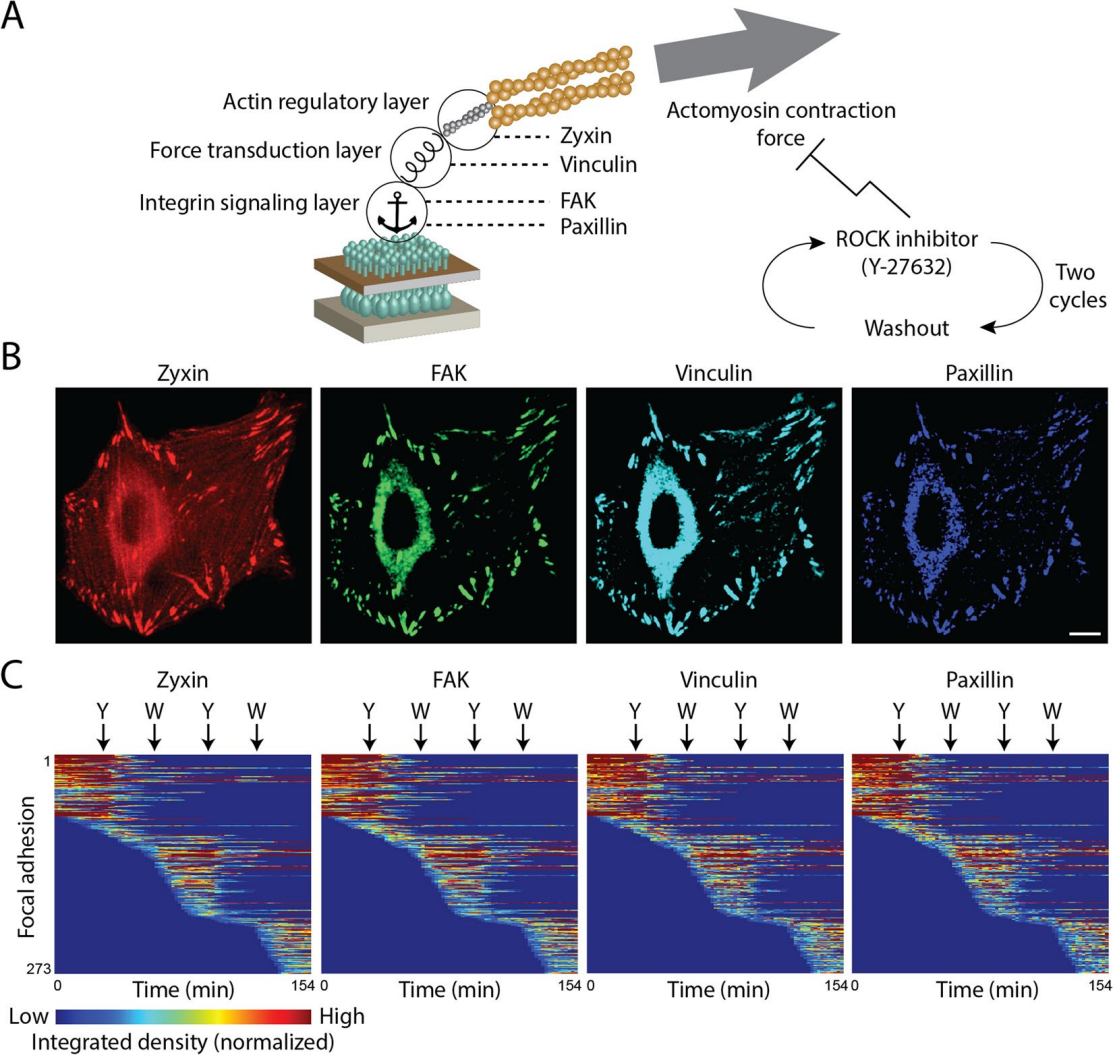
Four-color live cell imaging of focal adhesions responding to ROCK perturbations. (**A**) A scheme indicating the four examined components and their vertical positions within the focal adhesion^28^. Inhibition and recovery of actomyosin contractility were achieved by the addition and washout of the ROCK inhibitor Y-27632, respectively. (**B**) Images of a cell expressing TDmKate2-zyxin, mCitrine-FAK, TDmTFP-vinculin and TDmTagBFP-paxillin before the perturbations. Scale bar, 10 *μ*m. (**C**) Heatmaps show the integrated density of zyxin, FAK, vinculin and paxillin in each of the tracked focal adhesions of one cell as a function of time. Corresponding rows in the four heatmaps relate to the same focal adhesion. Arrows indicate the time of Y-27632 (Y) additions and washouts (W). The anchor image was obtained from Wikimedia Commons.

To facilitate sensitive 4-color live cell imaging of focal adhesions, we selected an optimal genetic-tagging combination of four fluorescent proteins, based on their photostability and spectra – mTagBFP, mTFP, mCitrine and mKate (Supplementary Fig. S1). Although this combination maximizes the spectral separation, inevitable partial spectral overlaps between the four fluorescent proteins still exist. Sequential excitation and imaging of each fluorophore facilitates further the spectral separation, without reducing the desired acquisition rate of one image every two minutes. Still, to completely separate the signals of the four fluorescent proteins it is required to narrow down the range of wavelengths collected for each one, which reduces the signal-to-noise ratio^19^. To compensate for this, we used tandem repeats (TD) of three of the fluorescent proteins (TDmTagBFP, TDmTFP, TDmKate2), thereby increasing the total brightness of these labels (Supplementary Fig. S1). An undesirable side-effect of such brightness increase can be a higher bleed-through to the other channels. However, since the edges of the emission spectra decay steeply and non-linearly along the spectrum, it is possible to effectively omit such bleed-through by narrowing down slightly the range of wavelengths collected for each fluorophore (Supplementary Fig. S1). The gain of brightness increases the signal-to-noise ratio, thereby enabling quantitative imaging in live cells of four proteins in the same small structures, such as cell-matrix adhesion sites (Fig. 1).

### Heterogenous responses of focal adhesion components to ROCK perturbations

**Figure 2:**
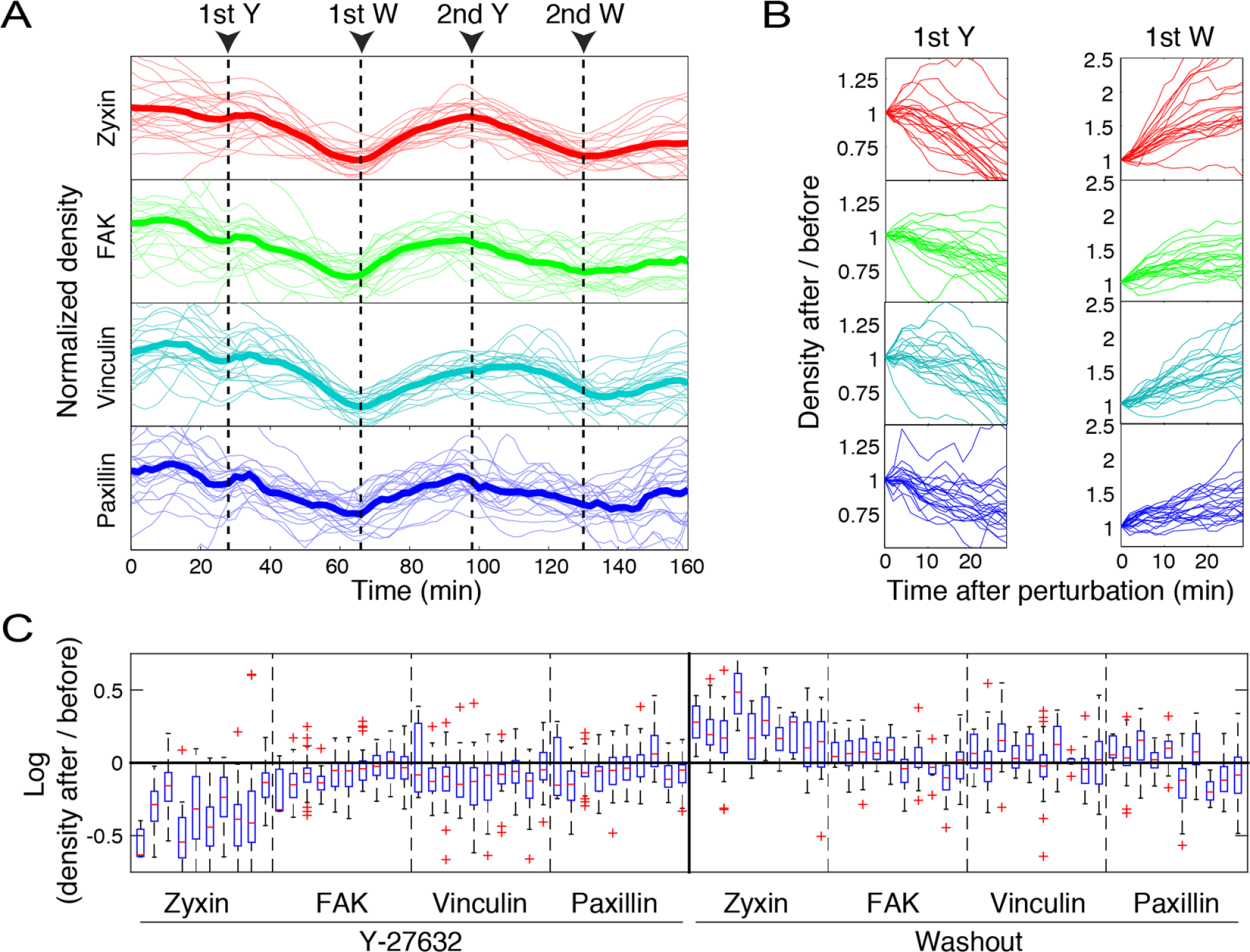
Diverse response extents of focal adhesion components upon ROCK perturbations. (**A**) The densities of zyxin, FAK, vinculin and paxillin in single focal adhesions of one cell are shown as a function of time along the experiment. Only focal adhesions that existed throughout the whole imaging period are shown. Each trace was normalized by dividing its values by their mean. Thick lines denote the traces average (*n* = 22 focal adhesions) at each time point. Arrows indicate the time points of Y-27632 additions (Y) and washouts (W). (**B**) The density changes of the same focal adhesions shown in (**A**), partitioned to the time periods following each perturbation. The intensity values of each trace segment were divided by its first value, to illustrate the fold change in density. (**C**) Box plots of the response extents of zyxin, FAK, vinculin and paxillin upon each perturbation in the focal adhesions of each of the ten analyzed cells (ten box plots within each dashed-line partition). The “before” and “after” density values were averaged along 3 sequential time frames before the perturbation and at 24 minutes after the perturbation, respectively. In the box plots, boxes indicate the interquartile range (IQR) between the first and third quartiles and the line within indicates the median. Whiskers denote the lowest and highest values within 1.5$\ast$IQR from the first and third quartiles, respectively. Crosses indicate data points beyond the whiskers.

For studying the molecular diversity in the mechano-responsiveness of focal adhesions we focused on four key proteins, including: paxillin and FAK which are vertically located within focal adhesions close to the plasma membrane, in the integrin signaling layer^27–31^, vinculin which is located at a higher vertical distance at the force transduction layer^28,32–35^ and zyxin which is embedded at the actin modulation layer and has been depicted as a prominent mechano-responsive component^13,14,28,36^ (Fig. 1A,B). Acute inhibition of actomyosin contractility was achieved by addition of the ROCK inhibitor Y-27632^21,37,38^ and recovery of contractility was achieved by washout of the drug (Fig. 1A). To investigate how mechano-responsiveness of focal adhesions is modulated by previous force perturbations, we applied two sequential cycles of ROCK inhibition and recovery (Fig. 1A,C). As expected, focal adhesions gradually disassembled upon actomyosin inhibition and reassembled following the washout of the inhibitor (Fig. 1C). The adhesion sites that were quantified were verified to be indeed focal adhesions, based on their zyxin content (Supplementary Fig. S2) and disassembly in response to ROCK inhibition (Fig. 1C). Most focal adhesions disassembled to sub-detection level following each of the Y-27632 treatments, while others disassembled partially and thereby spanned throughout the experiment (Figs 1C and 2A). Hence, the obtained data enables comparing the dynamic response patterns of zyxin, FAK, vinculin and paxillin to actomyosin contractility perturbations among a large number of single focal adhesions in live cells.

In order to assess the molecular diversity in the mechano-responsiveness of focal adhesions we quantified the densities (focal adhesion total intensity/area) of zyxin, FAK, vinculin and paxillin in single focal adhesions along the experiment (Fig. 2A,B). We calculated the response extent of a protein in a focal adhesion as log (density after perturbation/density before perturbation). This unit-less measure is independent of fluorophore brightness and detection efficiency, thus it enables quantitative comparisons between the responses of different proteins. Upon the first addition of ROCK inhibitor, the densities of zyxin, FAK, vinculin and paxillin decreased in most focal adhesions (Fig. 2C). Yet, the response extents of these proteins exhibited considerable intra-cellular and inter-cellular variabilities. Upon the first washout of the ROCK inhibitor, the density of zyxin increased in the vast majority of focal adhesions of all the analyzed cells, though with large intra-cellular variability (Fig. 2C). The response extents of FAK, vinculin and paxillin to the inhibitor washout exhibited considerable inter-cellular variabilities, and were sometimes also qualitatively distinct (Fig. 2C). The inter-cellular variability in the response extents of focal adhesions to this perturbation and to the followup perturbations can reflect differential recovery rates, at the cellular level, from a former perturbation. Yet, in comparison to the density of the proteins, their total level within each focal adhesion (indicated by integrated density per focal adhesion), decreased upon ROCK inhibition and increased upon washout more robustly (Supplementary Fig. S3). This robustness is plausible because the integrated density parameter integrates the area of the focal adhesion and the protein density within it, both of which typically change in the same direction in response to a mechanical perturbation. Since changes in the size of a focal adhesion affect the integrated density of all proteins, in order to differentiate between the responses of zyxin, FAK, vinculin and paxillin we focus hereafter on their density, rather than integrated density, changes. In general, the response extents of zyxin to ROCK perturbations were more pronounced than that of FAK, vinculin and paxillin (Fig. 2C and Supplementary Fig. S3), consistently with previous studies depicting zyxin as the strongest responder in focal adhesions to actomyosin perturbations^13,36^. However, previous studies in live cells were based mainly on examining one protein per cell, therefore could compare only the average responses of the different proteins. Here, we co-monitored zyxin FAK, vinculin and paxillin in the same focal adhesions, which enables to compare their responses in single focal adhesions rather than their averages.

### Diverse compositional changes in focal adhesions in response to ROCK perturbations

**Figure 3:**
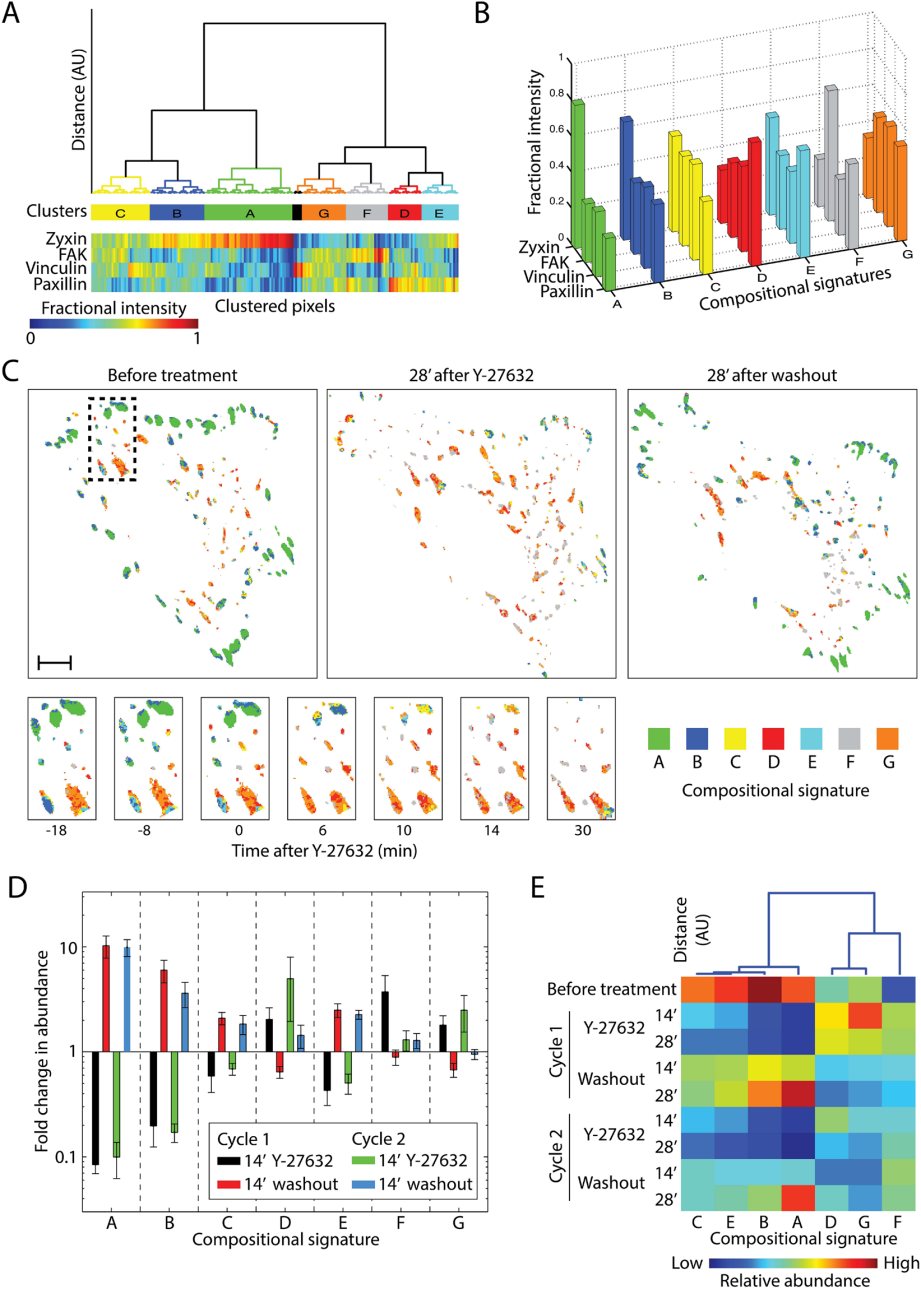
Compositional imaging analysis of focal adhesions upon ROCK perturbations. (**A**) Pixels of the 4-color live cell imaging of 5 cells clustered according to the fractional intensities of zyxin, FAK, vinculin and paxillin. (**B**) Bar plots presenting the average fractional intensity for each component in each cluster, defining the compositional signatures. (**C**) Compositional images of a cell (top) and of the marked (dashed line) region within it magnified (bottom) before and after the first Y-27632 addition. Pixels are colored according to their compositional signatures color-code, as shown in (**B**). Scale bar, 10 *μ*m. (**D**) Bar plots showing the mean of the fold change (14 minutes after perturbation/before perturbation) in the number of pixels (abundance) assigned to each compositional signature (*n* = 5 cells, error bars indicate standard error of the mean). (**E**) The compositional signatures clustered based on their abundance profile after the various perturbations. The heatmap shows the mean abundance of each composition at the indicated time points after ROCK perturbations (*n* = 5 cells). The columns of the heatmap are ordered according to the hierarchical clustering dendrogram shown on top.

We asked whether the observed variability in the response extents of zyxin, FAK, vinculin and paxillin to ROCK reflects different changes in the compositions of focal adhesions. To resolve and visualize compositional changes from multicolor data it is important to consider all the labeled components simultaneously. Compositional imaging achieves this by clustering the pixels of multicolor images according to the similarity in the intensity ratios between the labeled components^17^. Here we extended compositional imaging to multicolor live cell data (see Methods) and thus resolved 7 distinguishable compositional signatures within the focal adhesions during their responses to ROCK perturbations (Fig. 3A,B). Visualizing the localization of these compositions in the cells revealed differential spatiotemporal organizations (Fig. 3C, Supplementary Fig. S4 and Supplementary Movies S1–S5). Compositions which are enriched with zyxin (named A, B, C and E) get less abundant upon Y-27632 additions and more abundant upon washouts (Fig. 3B–E and Supplementary Movies S1–S5). In contrast, compositions with low levels of zyxin (D, F and G) become more abundant upon Y-27632, while getting less abundant upon its first washout with the exception of composition F (Fig. 3B–E). Interestingly, while changes in the abundance of zyxin-rich compositions (A, B, C and E) were consistent among the two perturbation cycles, changes in the abundance of zyxin-low compositions (D, F and G) were not (Fig. 3D). This points toward a potential modulation of the focal adhesion mechano-responsiveness by past mechanical perturbations and depicts zyxin as a component which is less sensitive to such modulations.

Peripheral focal adhesions were typically enriched with compositions A and B, having relatively high levels of zyxin (Fig. 3C), consistently with our previous findings in fixed cells^17^. Imaging the dynamics of these focal adhesions, indicates that they disassemble almost entirely upon Y-27632 addition (Fig. 3C and Supplementary Movies S1–S5). In contrast, central focal adhesions contained more of compositions D and G (low zyxin levels) and remained rather stable after ROCK inhibition (Fig. 3C and Supplementary Movies S1–S5). Consistently, enrichment of relative zyxin levels in the pre-perturbation composition of focal adhesions was overall correlated with a higher decrease in the integrated density of zyxin, FAK, vinculin and paxillin upon ROCK inhibition (Supplementary Fig. S5). The changes in the area of focal adhesions and in the density of FAK, vincuin and paxillin were variable, and overall differed only marginally between zyxin-enriched and zyxin-poor focal adhesions. Additional correlations were found between the area of focal adhesions and their compositions. In bigger focal adhesions, compositions with low zyxin/paxillin ratio (compositions D, E, F, G) are less abundant, while composition A, which has the highest zyxin/paxillin ratio, is more abundant (Supplementary Fig. S6). These findings are consistent with force traction microscopy studies, showing that peripheral focal adhesions, as well as large focal adhesions, are typically subjected to higher contractile forces than centrally located ones^39^. Upon inhibition of actomyosin contractility, a focal adhesion that was subjected to a higher force will experience a larger change in force levels, leading to larger response extents of its components. Based on these results, we conclude that the compositional changes occurring in focal adhesions upon ROCK perturbations are diverse and partially correlated with the location of the focal adhesions, their size and pre-perturbation molecular composition.

### Weak correlations between the responses of focal adhesion components to ROCK perturbations

**Figure 4:**
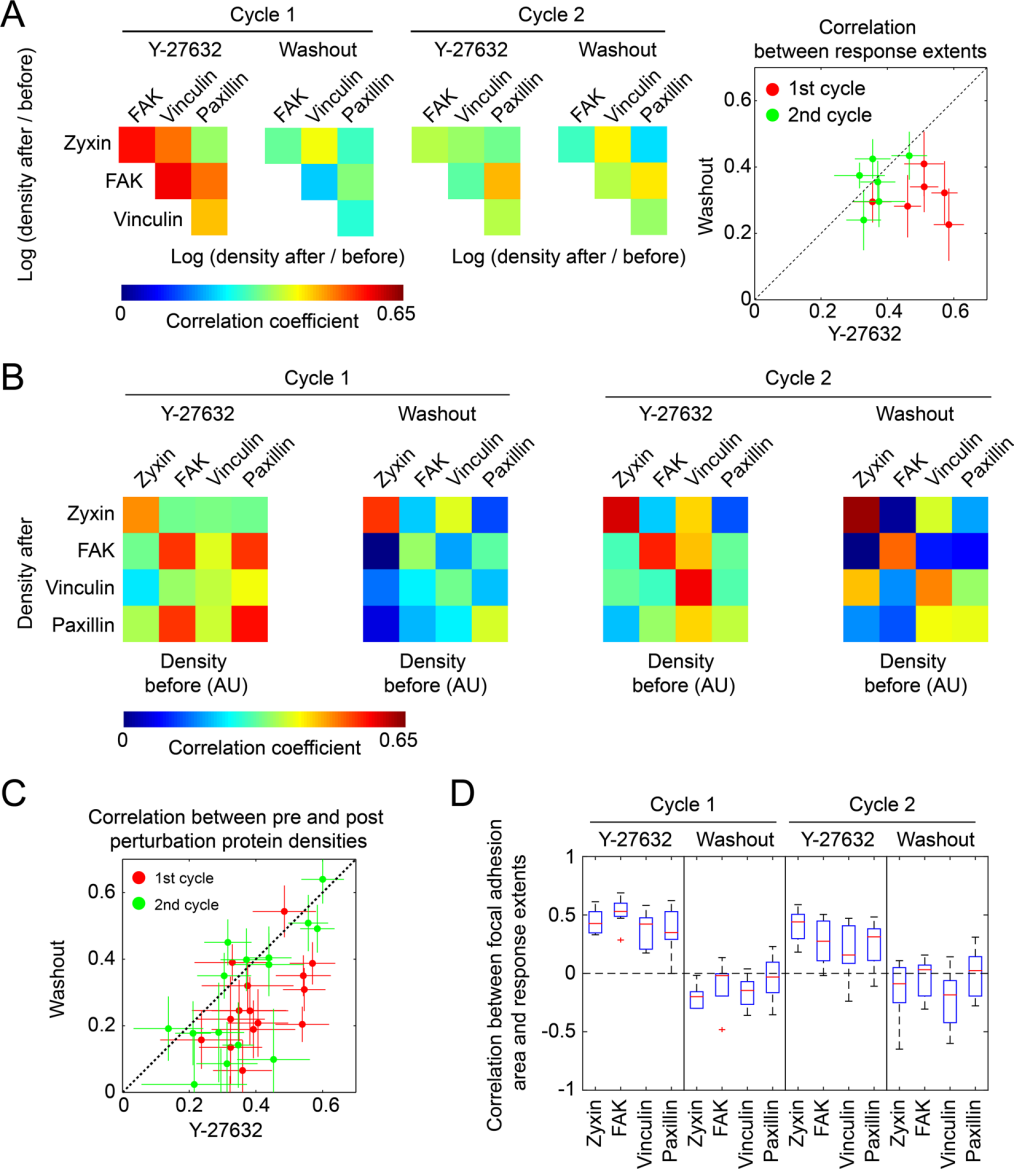
Correlations of the responses of zyxin, FAK, vinculin and paxillin to ROCK perturbations. (**A**) Heatmaps presenting the average Pearson correlation coefficients between the fold change in the density of each protein before perturbation and 12 minutes after perturbation. The scatter plot compares the average Pearson correlation coefficients between the Y-27632 and the washout treatments of the same perturbation cycle. Error bars indicate standard error of the mean (*n* = 10 cells, the number of focal adhesions per cell and perturbation spanned from 3 to 54 with an interpolated median of 27.5). (**B**) Heatmaps presenting the average Pearson correlation coefficients between the density of each protein before and 12 minutes after perturbation (*n* = 10 cells). (**C**) A scatter plot comparing the correlations of the pre- and post- perturbation protein densities between ROCK inhibition and washout treatments of the same perturbation cycle. Error bars indicate standard error of the mean (*n* = 10 cells). (**D**) A box plot of the correlations between the area of focal adhesions and the response extents of the examined proteins. Here, the response extent is calculated as log (density before/24′ after perturbation) for Y-27632 addition and as log (density 24′ after perturbation/before) for washout. For the sake of capturing robustly the trends, cases in which the number of focal adhesions per cell per perturbation was less than 20 were excluded from the analysis, which affected the variance of the data points but only marginally the medians (not shown), 9 ≥ *n* ≥ 6 cells.

The observed variability in the responses of proteins to ROCK perturbations can be driven by spatial diversity in local external cues (e.g. different force levels), diversity in the internal composition of focal adhesions and biochemical or measurement noise^15^. Random noise is expected to lead to response extents which are uncorrelated between the proteins, while diversity in biological cues can give rise to correlated responses^15^. The pairwise correlations between the response extents of zyxin, FAK, vinculin and paxillin across focal adhesions were found to be significantly positive (Fig. 4A and Supplementary Fig. S7). Therefore the observed variability in the response extents is not dominated by random measurement noise or biochemical noise. Potential correlated noise between the imaging channels is also not expected to affect the derivations of response extents, as for these calculations the densities of proteins before and after perturbations were averaged over three sequential time points. Therefore the observed variability indicates, at least in part, biological diversity which is plausibly driven by variability of local cues.

While the correlations between protein responses to ROCK perturbations are significantly positive, the strengths of these correlations are moderate or weak, with average Pearson correlation coefficients ranging between 0.2–0.6 (Fig. 4A). Of note, the correlations between protein responses to the first addition of Y-27632 were stronger than the corresponding correlations upon the followup perturbations (Fig. 4A). Yet, this can reflect the overall stronger responses of the focal adhesions to the first perturbation. Pair-wise comparisons of the response extents of proteins indicate that they can be approximated to be either statistically independent or linearly related (Supplementary Fig. S7). Therefore, although different proteins may have different, nonlinear, response properties to force and other cues, the lack of strong correlations between their responses is unaccountable by deviation from linearity. Instead, the weak or moderate correlations between the responses of the proteins to ROCK perturbations suggest that additional multiple local and independent cues shape these responses differentially.

### Differential relations between the mechano-responsiveness of focal adhesion components and local cues

We next sought to asses the statistical relations between the response extents of zyxin, FAK, vinculin and paxillin with various local cues of focal adhesions. An important aspect which can affect the response of a focal adhesion to a perturbation is its own internal molecular state. Therefore, a possible source for the variable response extents of proteins to force is the heterogeneous molecular content of focal adhesions before the perturbation is applied^16,17^. Moreover, the internal state of the focal adhesion reflects also the local cues affecting it, which can be spatially heterogenous. The obtained four-color live cell imaging data enable examining how the pre-perturbation densities of zyxin, FAK, vinculin and paxillin relate with their post-perturbation densities in single focal adhesions. The statistical relations between the densities before and after perturbations can be approximated as linear (not shown). The densities of each protein before and 12 minutes after a given perturbation are positively correlated at a variety of strengths, having average Pearson correlation coefficients up to around 0.6 (Fig. 4B and Supplementary Fig. S8). Almost all of these correlations are significantly positive, based on having mean values larger than the corresponding standard error of the mean (*n* = 10 cells; Fig. 4C and Supplementary Fig. S8A). Similarly, many of the differences between the correlation strengths (Fig. 4B) are statistically significant, based on their standard error of the mean (Fig. 4C and Supplementary Fig. S8). As expected, the correlations of the pre-perturbation densities of a protein with its density after 12 minutes are stronger than with 24 minutes after perturbation (Supplementary Fig. S8A). The decrease in these correlations with time reflects both variable rates of density changes among focal adhesions as well as convergence toward different steady-state levels of density. Moderate or weak positive hetero-protein correlations were found also between the pre-perturbation density of a given protein and the post-perturbation density of another protein (Fig. 4B). Intriguing exceptions are the relatively strong hetero-protein correlations of the pre-perturbation densities of FAK and paxillin with the densities of paxillin and FAK after the first addition of Y-27632, respectively (Fig. 4B). These correlations can be due to the release of FAK-paxillin as a complex from disassembling focal adhesions^40,41^. In contrast, although vinculin and paxillin were also reported to be released as a protein complex from focal adhesions^40,41^, the correlation between their pre- and post- densities are weaker than between FAK and paxillin (Fig. 4B). Hence it is plausible that disassembly of focal adhesions triggered by acute inhibition of contractility is a molecularly distinct process than their disassembly due to steady-state turnover of focal adhesions in spread cells. Almost all the correlations between pre-perturbation and 12-minutes post-perturbation protein densities were found to be stronger in the case of Y-27632 in comparison to washout, particularly in the first perturbation cycle (Fig. 4B,C). This could be explained by a first-order disassembly kinetic of the focal adhesions upon actomyosin inhibition, such that proteins and complexes are released at rates which are proportional to their densities in the structure. In contrast, the recruitment rates of proteins to focal adhesions upon actomyosin recovery are plausibly dominated more by the strength of the force applied on each focal adhesion as well as other cues, and are therefore less affected by the pre-perturbation density of proteins in these structures. Interesting exceptions are the correlations between the pre-perturbation and post-perturbation densities of zyxin, which were strong, and not weaker upon the washouts in both perturbation cycles, in comparison to the corresponding Y-27632 addition (Fig. 4B). This suggests that zyxin has a positive influence on its own recruitment to focal adhesions upon increase of contractile forces.

We next examined whether the response extents of zyxin, FAK, vinculin and paxillin to the ROCK perturbations correlate with the pre-perturbation size of focal adhesions. In focal adhesions which are bigger before perturbation, the response extents of the proteins to ROCK inhibition tend to be bigger, though with a moderate correlation strength (Fig. 4D). In comparison, the areas of focal adhesions before Y-27632 washout were more weakly correlated with the response extents of zyxin, FAK, vinculin and paxillin upon the washout (Fig. 4D). A possible explanation for this asymmetry is that in the presence of unperturbed actomyosin contractility, the area of focal adhesions is more governed by the applied mechanical force, therefore bigger focal adhesions are likely to experience a bigger decrease in force levels upon actomyosin inhibition. In contrast, after the actomyosin contractility is inhibited for sufficient time the remaining size of the disassembled focal adhesions is plausibly determined by other factors which are not indicative of the expected elevation in force upon contractility recovery.

**Figure 5:**
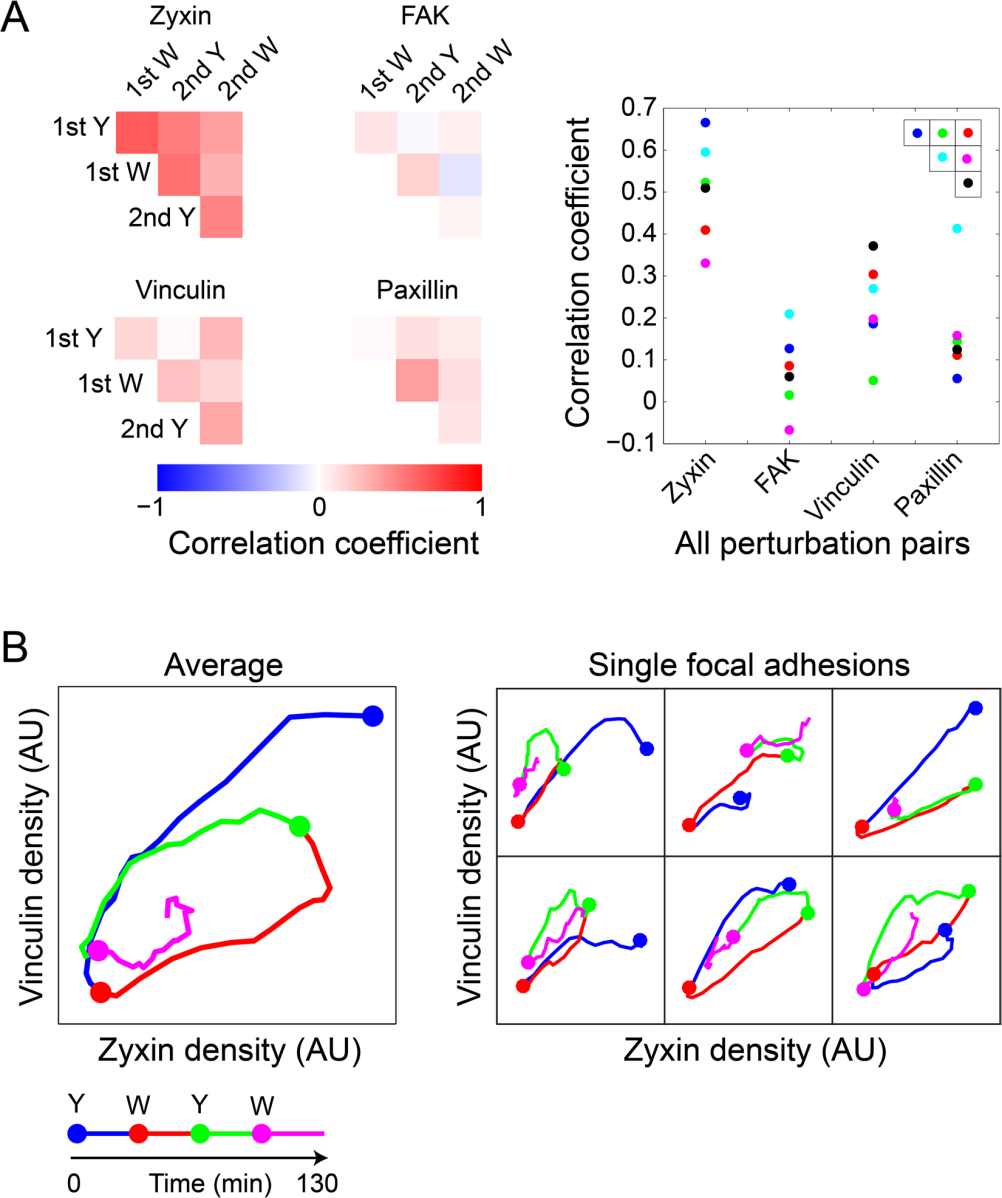
Diverse patterns of molecular changes among single focal adhesions in response to repeated ROCK perturbations. Focal adhesions which were present throughout both ROCK perturbations cycles were identified in 6 selected cells (*n* = 58 focal adhesions). (**A**) Heatmaps of the Pearson correlation coefficients between the strengths of the responses of each protein to the different perturbations. The response strengths to Y-27632 (Y) and washout (W) were calculated as log density (before/14′ after perturbation) and log density (14′ after perturbation/before), respectively. The plot shows the values of the correlation coefficients, color coded according to their positions in the corresponding heatmaps. For robust quantification of responses per individual focal adhesions, values of 3 sequential time frames were averaged before and at 14 minutes after each perturbation. (**B**) Phase-space plots of the densities of zyxin versus vinculin along the experiment, either averaged over the single focal adhesions (Left, *n* = 58) or for six single focal adhesions from one cell (right). The time-points of Y-27632 (Y) additions and washouts (W) are indicated color-coded by the filled circles.

Focal adhesions are often subjected to altering mechanical forces due to the dynamic reorganization of the actin cytoskeleton and the tension distribution throughout it^42,43^ as well as due to the dynamic modulation of the matrix^6,44^. Therefore, beside sensing the mechanical force applied at a given moment, it could be that focal adhesions are designed to assimilate also information about former changes in force they recently experienced. This possibility is consistent with the aforementioned differences of the responses of the proteins and their correlation patterns between the first and second ROCK perturbation cycles (Figs 3 and 4). In order to compare the differences between the responses of the same focal adhesions to the two repeats of ROCK inhibition and recovery, we examined the focal adhesions that existed throughout the two cycles of perturbations. The response extents of zyxin in these focal adhesions were found to be positively correlated along the four perturbations (Fig. 5A). Accordingly, in focal adhesions where zyxin had a stronger density decrease upon ROCK inhibition it also had a stronger density increase upon washout and so on continuously along the second perturbation cycle. In comparison to zyxin, the response extents of FAK, vinculin and paxillin exhibited overall weaker correlations between the perturbations (Fig. 5A). Overall, the correlations between the protein responses to the two Y-27632 additions, as well as between the responses to the two washouts, were weak. Therefore, not only that the responses of proteins to the second perturbation are different than their responses to the first perturbation, this difference varies between focal adhesions. This suggests that the response of the proteins in focal adhesions to ROCK can be modulated also by past perturbations, however the actual outcome of this modulation depends on other local parameters which vary between focal adhesions. Such an apparent memory could be achieved at the cellular state level, reflecting incomplete recovery from former perturbations. However, in addition it is possible that the protein network within a focal adhesion, and even individual mechanosensing proteins, are designed to have hysteresis or irreversibility in their steady-state changes upon force increase versus force decrease. An intriguing example for such irreversibility, emerging from the collective actions of two proteins, is the binding of vinculin to mechanically stretched talin, which inhibits the refolding of talin after the force is released^45^.

All the aforementioned correlation analyses were performed by sampling a single time point after each perturbation. Importantly, the values of these correlations are expected to change along the experiment, hence altering also the patterns of correlations. Indeed, comparing the correlations obtained for 12 minutes versus 24 minutes after perturbations, between pre- and post- perturbation densities, shows that not only they get weaker with time, they also change their relative magnitudes (Supplementary Fig. S8A). An additional possible bias is that a larger range of response extents can provide higher Pearson correlation coefficients with another parameter, since it is less affected by a given noise level. However, the important features that remain robust are the presence of significant, though moderate, correlations between the compared parameters, and that these correlations are often nonuniform among proteins, perturbation types and perturbation cycles. Therefore, taken together, the correlation analyses suggest that multiple local cues are integrated to shape differentially the responses of zyxin, FAK, vinculin and paxillin to actomyosin contractility perturbations.

### Diverse patterns of responses to ROCK perturbations among single focal adhesions

Given the variable responses of zyxin, FAK, vinculin and paxillin to ROCK perturbations, and the overall moderate correlations between them, we sought to discriminate between two alternative options regarding the path of the occurring molecular changes. Such a path can be described as a trajectory in a multidimensional phase space, where each dimension corresponds to the level of one of the imaged components, indicating the levels of the four proteins along the experiment. The first possibility is that the molecular changes that occur in different focal adhesions follow different quantitative manifestations of qualitatively the same path trajectory. This possibility implies that certain qualitative features of the trajectory shape should be retained in all responding focal adhesions. The alternative possibility is that in different focal adhesions the trajectory of the molecular changes has a qualitatively different shape. The difference between the two options is fundamental, since the first one implies a fixed pattern of molecular changes while the second option allows for flexible patterns of molecular changes to occur in response to ROCK perturbations. Two-dimensional phase space diagrams, showing for individual focal adhesions the levels of two proteins versus each other along the experiment, capture illustratable projections of response paths, in all possible pairwise protein combinations (Fig. 5B and Supplementary Fig. S9). The average path of molecular changes reflects the observed average response properties, including the stronger response of zyxin to the ROCK perturbations (Fig. 5B, left). However inspecting the paths of molecular changes in single focal adhesions uncovered a variety of qualitative differences in their trajectories (Fig. 5B, right). Hence, these observations indicate directly that the patterns of molecular changes occurring in focal adhesions are flexible to a considerable extent.

**Figure 6:**
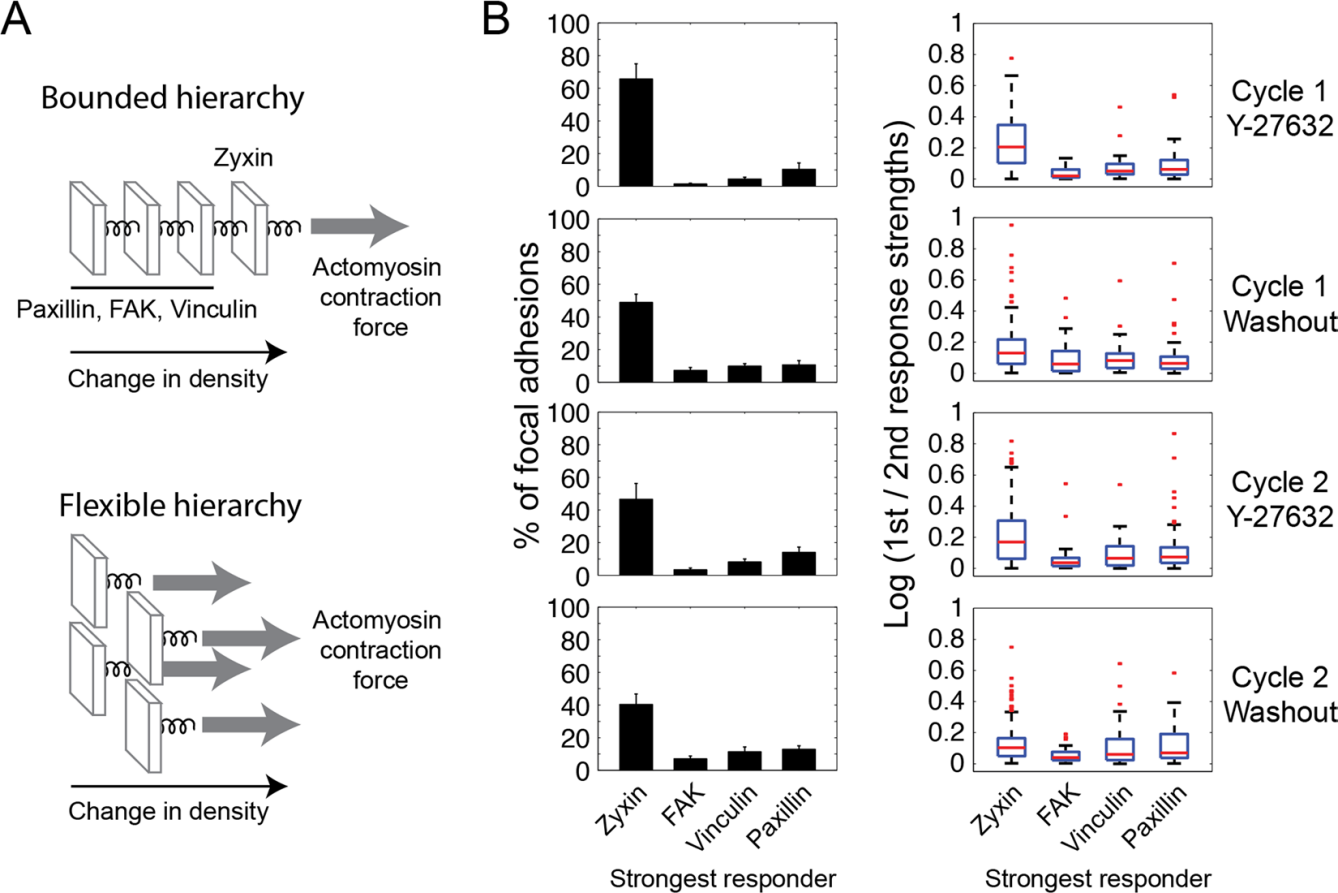
Diverse hierarchies of response extents of zyxin, FAK, vinculin and paxillin to ROCK perturbations. (**A**) A bounded hierarchy imposes a fixed order between the response extents of the proteins, metaphorized here as components linearly connected by springs, so that no component can be pulled more than the component on its right side. A flexible hierarchy enables different proteins to be the strongest responders, illustrated as components connected in parallel to the pulling force. (**B**) The bar plots (left column) show the percentages of focal adhesions having the indicated stronger responder upon each perturbation. A protein was counted as a stronger responder only if its response is at least 10% bigger than the responses of the other three proteins. Error bars indicate standard error of the mean (*n* = 10 cells). The box plots (right column) show the ratio between the strongest response and the second strongest response in focal adhesions having the indicated strongest responder. The response strength is calculated as log (density before/14′ after perturbation) for Y-27632 addition and as log (density 14′ after perturbation/before) for washout. In all box plots, boxes indicate the interquartile range (IQR) between the first and third quartiles and the line within indicates the median. Whiskers denote the lowest and highest values within 1.5$\ast$IQR from the first and third quartiles, respectively. Red dots indicate data points beyond the whiskers. For robust quantification of responses per individual focal adhesions the values of 3 sequential time frames were averaged before and at 14 minutes after each perturbation.

One interesting aspect of a response pattern is the hierarchy of response extents of the four proteins, namely which protein exhibits the biggest fold change, which one is the next stronger responder and so on. Accordingly, we asked whether these hierarchies are the same in all responding focal adhesions. If the extents of FAK, vinculin and paxillin responses are designed to be driven and bounded by the extent of zyxin response, then zyxin should be the strongest responder in every single focal adhesion (Fig. 6A, top). Alternatively, if zyxin, FAK, vinculin and paxillin are allowed to respond to ROCK through partially independent pathways then the identity of the strongest responder can vary among focal adhesions (Fig. 6A, bottom). To discriminate between these possibilities we sorted for each focal adhesion the fold changes of zyxin, FAK, vinculin and paxillin density, then counted the occurrence of each response extent hierarchy in the focal adhesions (Supplementary Fig. S10A). Strikingly, although zyxin was most abundantly the stronger responder, in a significant fraction of focal adhesions paxillin or vinculin were the strongest responders to ROCK perturbations (Fig. 6B and Supplementary Fig. S10B). In some focal adhesions FAK also exhibited the strongest response extent, yet less frequently in comparison with zyxin, vinculin and paxillin. In focal adhesions in which zyxin was the strongest responder, its responses were 25–100% higher than that of the next strongest responder (Fig. 6B). In contrast, in cases where either paxillin, FAK or vinculin were the strongest responder they responded up to 25% more prominently than the next strongest responder (Fig. 6B). Interestingly, systematic proteomic examinations indicated that the level of FAK is indeed less affected by actomyosin inhibition in comparison to vinculin and paxillin, and that vinculin and paxillin are less affected in comparison to zyxin^46,47^. Hence, the frequency by which zyxin, FAK, vinculin or paxillin arises as the strongest responder to ROCK in single focal adhesions correlates with their average sensitivity to actomyosin inhibition in a population of focal adhesions. These results explain why zyxin was found to be on average the strongest responder to ROCK perturbations, however they also reveal that in individual focal adhesions the identity of the stronger responder is actually distinct.

We next compared the abundances of the response extent hierarchies among the various ROCK perturbations. The abundances of these hierarchies were found to be positively correlated between the two repeats of the same kind of perturbation (Supplementary Fig. S10C). Therefore, the first cycle of perturbations did not alter the relative sensitivities of zyxin, FAK, vinculin and paxillin to actomyosin contractility with respect to each other. The abundances of the fold response hierarchies upon Y-27632 addition are strongly negatively correlated with those upon washout (Supplementary Fig. S10C). Since fold density change is calculated as log (density after/before perturbation) for all perturbations, these strong negative correlations indicate that the opposing force perturbations only rarely lead to opposed hierarchies of response strengths. Instead, the tendency of a given protein to arise as a stronger responder is comparable for ROCK inhibition and recovery, as reflected also in the distribution of the strongest responders (Fig. 6B). Taken together, the results show that the hierarchy of zyxin, FAK, vinculin and paxillin response extents to ROCK perturbations, as well as the overall dynamic response pattern, are not conserved but instead significantly varying among single focal adhesions.

## Discussion

**Figure 7:**
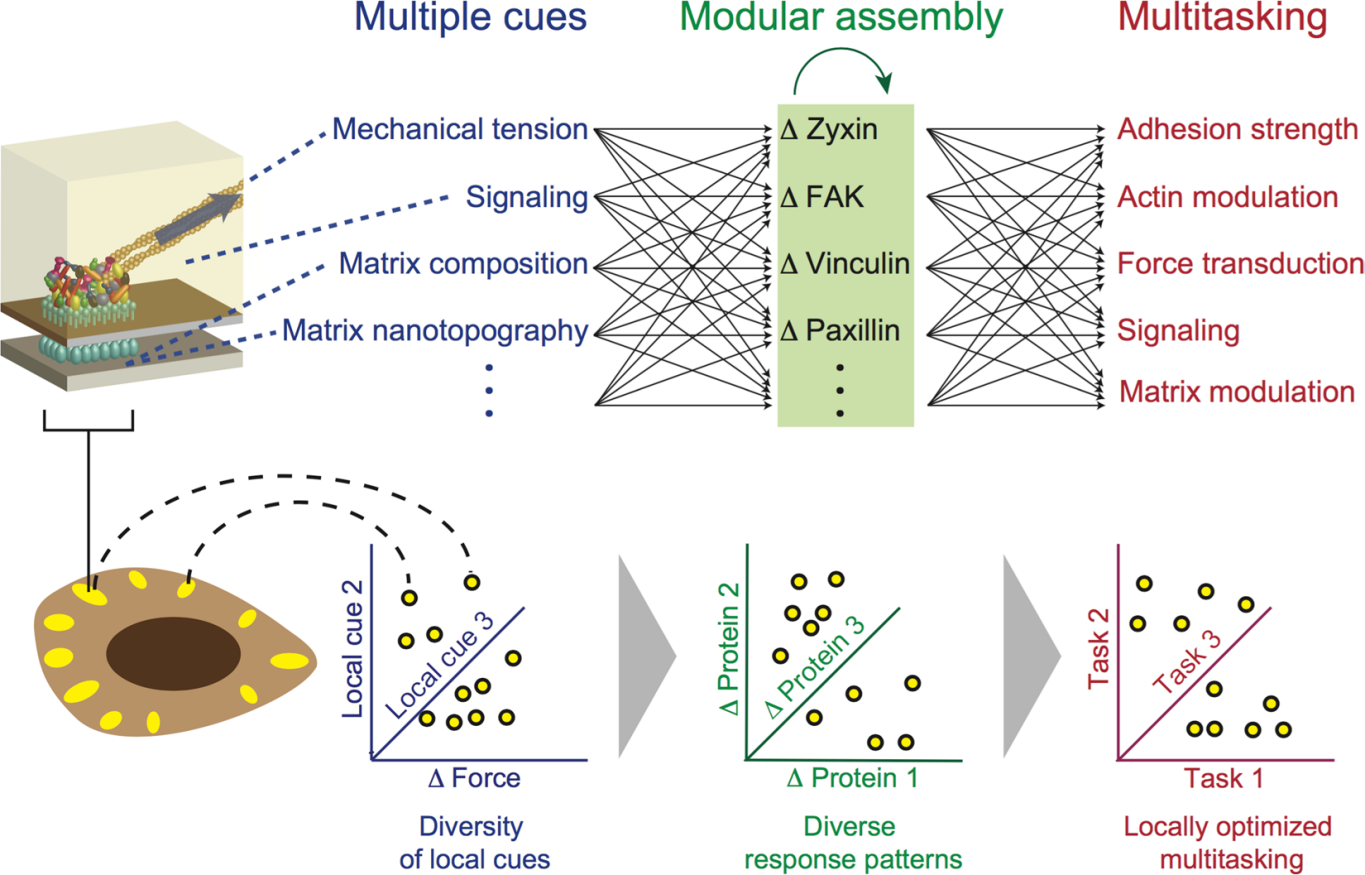
A model for the molecular diversity in the mechano-responsiveness of focal adhesions and its coupling with their modular mode of assembly and multitasking. The molecular diversity in the responses of focal adhesions to actomyosin perturbations is attributable to multiple factors, including their pre-perturbation internal state, the local change in force level, past changes in force levels, local signaling within the cell and local matrix properties. Hence, assimilation and encoding of these spatially varying cues by each focal adhesion cause distinct patterns of molecular changes in different focal adhesions. The modular mode of focal adhesions assembly enables the shaping of the response patterns, as it allows changing the stoichiometric ratio between the components within a focal adhesion. In turn, the flexibility in setting the response patterns can enable a focal adhesion to adjust the proportions between its multifunctional components, so that collectively they perform correctly its multiple tasks given its local cues.

Cell-matrix adhesion sites perform their diverse functions via dynamic changes in their internal molecular organization. Focal adhesions sense and respond to mechanical forces via changing their morphology and molecular composition. Intriguing questions are how these changes incorporate additional cues that focal adhesions need to sense and how are they concerted with the additional tasks of focal adhesions. In order to address these questions, it is important to observe the patterns of molecular changes in individual focal adhesions, rather than inferring an average pattern from the changes of single proteins in different focal adhesions. Here we achieved this, by applying for the first time 4-color live cell imaging of focal adhesions responding to ROCK perturbations. This imaging uncovered previously inaccessible diversity in the molecular events occurring in focal adhesions in response to actomyosin contractility. Importantly, we found that not only the response extents of each protein to ROCK vary, but also the combined pattern of zyxin, FAK, vinculin and paxillin responses is different among single focal adhesions. The observed moderate or weak correlations of protein response extents with other factors suggest that the multi-dimensional diversity in the mechano-responsiveness of focal adhesions reflect integration and encoding of multiple local cues (Fig. 7, top panel). A notable manifestation of the diversity in the mechano-responsiveness of focal adhesions concerns zyxin – a component which is considered to be tightly coupled with actomyosin contractility. Unlike paxillin, vinculin and FAK, zyxin is absent in focal complexes and gets initially recruited only once these structures mature to focal adhesions and become associated with contractile stress fibers^48,49^. Additionally, it was shown by imaging and proteomic approaches that on average over a population of focal adhesions zyxin exhibits the strongest response to force perturbations, in comparison to the other examined components^13,14,36,46,47,50^. While our findings are consistent with the previous studies, we reveal here by 4-color imaging that in different focal adhesions different proteins emerge as the strongest responders to force perturbations.

We hypothesize that the diversity in the patterns of molecular changes in focal adhesions in response to contractility reflects a fundamental engineering principle enabling these structures to achieve multitasking. According to this hypothesis, the modular mode of focal adhesions assembly enables them to receive and encode multiple cues, in addition to the mechanical tension level, in their internal molecular organization. Such input cues plausibly include the local chemical and topographic properties of the matrix, the location of the focal adhesion within a cell and former force changes. Thus, spatial and temporal diversity in these local cues within the cell can generate different patterns of molecular events in focal adhesions responding to actomyosin perturbations, as we observed (Fig. 7, bottom panel). Upon the integration of its multiple cues, the focal adhesion needs to respond by adjusting its mechanical and signaling properties (Fig. 7, top). Although some focal adhesion components are associated more with mechanical aspects, while others with signaling aspects, most components contain multiple domains which are involved with the variety of focal adhesion signaling and mechanical activities. Furthermore, the network of possible interactions between the components of focal adhesions implies that each component can affect multiple aspects of these structures, either directly or by affecting other components. Hence, an arising question is how can a focal adhesion perform optimally its multiple tasks using a common set of components, each of which affects differentially a variety of tasks. In contrast to an assembly mode in which each component can be recruited to a structure in one defined way, focal adhesions are assembled in a modular mode as their components can interact in many alternative manners. We suggest that such a modular mode of assembly provides the focal adhesion an essential degree of freedom to adjust the level of each component relatively independently of the other components. Thus the focal adhesion can achieve a combination in which each of its components is at the right level, so that collectively they perform optimally the variety of focal adhesion tasks, in addition to mechanosensing.

This work indicates that the mechano-responsiveness of focal adhesions should be conceived and investigated as a molecularly diverse process, as it consists of heterogenous patterns of multi-protein changes. While we identified several factors, each of which having a detectable correlation with the responses of proteins, deciphering how exactly their patterns are shaped by the multiple cues is a major goal to further address. The fundamental challenges to achieve this deciphering are the large number of potential cues and the huge dimensionality of the response patterns – as each of the tens proteins located in focal adhesions adds a dimension. High-throughput implementations of multicolor live cell imaging, though technically challenging^19^, can provide a sufficiently large sample size for capturing high-dimensional statistical relations between the dynamic responses of multiple proteins. Systematic, and combinatorial, perturbations of specific cues would allow to gradually uncover how they collectively shape the patterns of molecular changes in the focal adhesions. Combining traction force microscopy measurements^4,8,51^, direct mechanical perturbations of individual focal adhesions^9^ and multicolor live cell imaging would be valuable for inferring the causal effect of force on the patterns of molecular changes in these structures. Synthetic, well defined, biointerfaces^2,52,53^ would allow to independently control a variety of matrix properties, thus studying how they influence the molecular changes in focal adhesions in response to force. While these aforementioned directions address the mechanisms underlying the diversity of the response patterns, analyses such as Pareto optimality can help understanding how these patterns serve the multitasking of focal adhesions and formulating these tasks at the molecular level^54^. At a higher functional level, the mechanosensitivity of focal adhesions enables the cell to sense and respond to the rigidity of the matrix, gradients of matrix rigidity^4^ and matrix topography^2^. Understanding the dynamics of protein networks in focal adhesions in response to actomyosin contractility would provide an important basis for studying higher cellular functions which are based on this mechanosensitivity.

## Methods

### Cell culture and transfection

Rat embryonic fibroblasts (REF52; kindly provided by Joachim Spatz and Benjamin Geiger) were cultured in Dulbecco’s modified eagle’s medium (DMEM) containing 10% fetal bovine serum (Invitrogen, Carlsbad, CA, USA), 1% penicillin/streptomycin and 1% L-glutamine (PAN Biotech GmbH, Aidenbach, Germany) in a 5% CO_2_ humidified incubator at 37 °C. For live cell imaging, cells were plated with 400 *μ*l DMEM in 8-well LabTek chamber slides (Thermo Fisher Scientific, Waltham, MA, USA) at a density of 10^4^ cells per well and co-transfected the next day with a tandem (TD) monomeric Kate2 (TDmKate2-zyxin)^40^, mCitrine-FAK^40^, TDmTFP-vinculin and TDmTagBFP-paxillin (constructed by Kondaiah Moganti and Ruth Lambertz née Stricker, Max Planck Institute of Molecular Physiology, Dortmund, Germany) using Lipofectamine 2000 (11668-019, Invitrogen). Following transfection, cells were incubated for 24 hours prior imaging.

### Microscopy

Multicolor live cell imaging was performed using a Zeiss LSM 510 Meta confocal microscope (Carl Zeiss, Jena, Germany) with a 40x water immersion objective and pre-warmed incubation chamber at 37 °C with 5% CO_2_. Before imaging, the growth media was replaced by imaging media (P04-01163, PAN Biotech, GmbH) supplemented with 10% fetal bovine serum. Multicolor imaging data was acquired by sequential excitation and detection of TDmKate2-zyxin (excitation at 561 nm and emission detection at 575–800 nm), mCitrine-FAK (excitation at 514 nm and detection at 530–565 nm), TDmTFP-vinculin (excitation at 458 nm and detection at 470–500 nm) and TDmTagBFP-paxillin (excitation at 405 nm and detection at 420–480 nm). At the indicated time points during the time lapse imaging, Y-27632 (Y0503, Sigma-Aldrich, St Louis, MO, USA) was added from a stock solution of 10 mM in water to give the final applied concentration of 100 *μ*M, to inhibit ROCK and later washed out from the cells dish using imaging media (“washout”). The applied dose of Y-27632 facilitates an abrupt and synchronized inhibition of actomyosin contractility^17,38,55–58^, thereby enabling to compare the dynamics of proteins across different focal adhesions and cells. For chromatic aberrations correction, multicolor TetraSpeck 1 *μ*m diameter beads (T14792, Invitrogen) were imaged using the same setup, but with a higher laser power to enhance the signal-to-noise ratio.

### Image preprocessing

The acquired multicolor live cell data consist of an array of images, *Img*^*raw*^(*c*, *t*, *p*), where *c* = 1, …, 10 cell index, *t* = 1, …, *n* time frame and *p* = 1, …, 4 imaging channel. Chromatic aberrations were measured based on the multicolor TetraSpeck beads images and accordingly each image in *Img*^*raw*^(*c*, *t*, *p*) was corrected with respect to its corresponding mCitrine channel image. The background intensity in each image was measured in a region outside cells and then subtracted from the whole image. Then bleaching was corrected by multiplying each image by a factor that keeps the total intensity of the corresponding protein in the corresponding cell constant along time. High-frequency spatial noise in these images was removed by low-pass mean filtration with a radius of one pixel. Then, low-frequency spatial background noise was removed by high-pass filtration in which the value of each pixel is subtracted by the mean intensity of the pixels within a 6 *μm*-wide box around it. Pixels with negative values were then set to zero. Lateral shifts between images of different time frames, due to minor movements of cell dish during acquisition and perturbations, were corrected by restoring the xy-shift in the pixels as estimated from the image registration of the reference (i.e. mCitrine) channel. For compositional imaging (Fig. 2), high-frequency noise was smoothed using the nsmooth algorithm^59^. For the analysis of single focal adhesions responding to two repeated perturbations (Figs 2A and 5) Kalman filtration was applied.

### Compositional imaging

Compositional imaging resolves and visualizes spatial and temporal differences in the molecular composition of intra-cellular structures^17^. Similar to ratio imaging between two proteins^6,16^, in compositional imaging the ratio between the levels of labeled and unlabeled copies of a component is an unknown constant which gets mathematically cancelled in the comparisons of compositions between time points or spatial positions^17^. For compositional imaging analysis pixels were pooled from the processed *Img*^*pr*^(*c*, *t*, *p*) images of five cells, resulting in an array $Px{l}^{all}(c,t,x,y,\vec{I})$ where *c* = 1, …, 5 cells, *t* = 1, …, *n* time frames, *x* and *y* indicate the pixel position, and $\vec{I}$ is a vector containing the intensity levels in the four imaging channels in this pixel. Pixels outside focal adhesions with background intensity levels were removed by setting an intensity threshold for each protein in each cell and excluding those pixels that have for all proteins intensity values lower than the corresponding thresholds, resulting in a shorter list of pixels. To make the data comparable between cells and to give each protein an equal weight in the further analysis, the intensity values of each protein were then normalized by dividing them by the median value of all pixels with positive intensity values in the corresponding cell. Pixels with overall low intensity values were excluded by thresholding them based on the root of the sum of squared intensity values of the four proteins, $\sqrt{({\rm{\Sigma }}{I}_{j}^{2})}$. The composition stoichiometry of the four proteins in each pixel was then calculated by dividing the intensity value of each protein by $\sqrt{({\rm{\Sigma }}{I}_{j}^{2})}$, thereby obtaining its fractional intensity. The resulted array of pixels were then clustered by hierarchical clustering with spherical distance metric to group pixels with similar composition using a custom C++ program. The program implements the standard hierarchical agglomerative clustering algorithm but calculates the distance between points over the surface of a unit sphere. New points resulting from the merge of others are also calculated over such a surface, to maintain the stoichiometric normalization of the data. To enable clustering large datasets, the program does not store in memory the distance between points but calculate them on demand. This results in a longer calculation time which is mitigated by applying a parallel computing approach implemented using OpenMP. The obtained clustering dendrogram was partitioned interactively to a set of clusters which are consistent with its topology and exhibit distinct spatial or temporal profiles. These clusters were spatially visualized as compositional images by color-coding each pixel according to the cluster it belongs to.

### Tracking focal adhesions and analysis

Each channel of the processed image series was thresholded and intensity values of pixels below the threshold were set to zero. Focal adhesions in thresholded images were then segmented using water algorithm^16^, implemented in Matlab (The MathWorks, Inc., Natick, Massachusetts, United States), to generate four segmented images for each time frame. In order to obtain a unifying segmented image for all four proteins in each time frame, intensities of pixels under each segmented focal adhesion were normalized by dividing them by their mean intensity. The four normalized segmented images were then summed together to generate a single image. These images were then segmented again using water algorithm to generate for each time frame a common segmented image for all four proteins. Using these common segmented images, focal adhesions were tracked by matching them between each consecutive time frames. This matching was based on the maximum overlap of their area, or on the shortest distance between their center of mass within a radius of 35 pixels if no area overlap was found. The obtained matches were then inspected interactively and corrected where needed. The integrated density of a protein in a focal adhesion at a given time point was calculated as the sum of the corresponding intensities of all pixels assigned to it at that time point. The density of each protein in a focal adhesion at a given time was calculated as its integrated density divided by the number of pixels assigned to this focal adhesion at that time point. To standardize data obtained from different cells, the intensity values of each protein in each focal adhesion and time point were divided by the sum of the accumulated intensity of this protein in all the focal adhesions in the given cell over all the time points. Beside of correlating protein response extents with pre-perturbation protein densities, the intensity trace of each protein in each focal adhesion was further normalized by dividing its sum. Unless otherwise indicated, protein response extents were calculated as log (density after perturbation/density before), a measure which cancels brightness differences between fluorescent proteins and different detection sensitivities at each imaging channel, therefore enabling comparing between the response extents of different proteins. In addition, this response extents derivation formula captures the fold change in the total density of a protein in a focal adhesion, since both the labeled and unlabeled protein copies have the same fold of change. Tracking and analyses of the obtained intensity traces were performed in Matlab (The MathWorks, Natick, MA, USA).

## Electronic supplementary material

Supplementary Movie S1

Supplementary Movie S2

Supplementary Movie S3

Supplementary Movie S4

Supplementary Movie S5

Supplementary Information
