# Supplementary material for: Diverse patterns of molecular changes in the mechano-responsiveness of focal adhesions
Source: Sci Rep. 2018 Feb 1;8:2187. doi: 10.1038/s41598-018-20252-0 (PMC5795008; doi:10.1038/s41598-018-20252-0)
Supplement: Supplementary file 6 — Supplementary Information [file 41598_2018_20252_MOESM6_ESM.pdf]

Supplementary Figures

Diverse patterns of molecular changes in the mechano-responsiveness of focal adhesions

Rahuman S. Malik-Sheriff[1,4,+], Sarah Imtiaz[1,+], Hernán E. Grecco[1,3], Eli Zamir[1,2,*]

[1]Department of Systemic Cell Biology, Max Planck Institute of Molecular Physiology, Dortmund, Germany.
[2]Department of Cellular Biophysics, Max Planck Institute for Medical Research, Heidelberg, Germany.
[3]Current address: Department of Physics, FCEN, University of Buenos Aires and IFIBA, CONICET, Argentina.
[4]Current addresses: European Bioinformatics Institute, European Molecular Biology Laboratory, Hinxton, Cambridge, UK.
[+]These authors contributed equally to this work
[*]Corresponding author: eli.zamir@mpimf-heidelberg.mpg.de

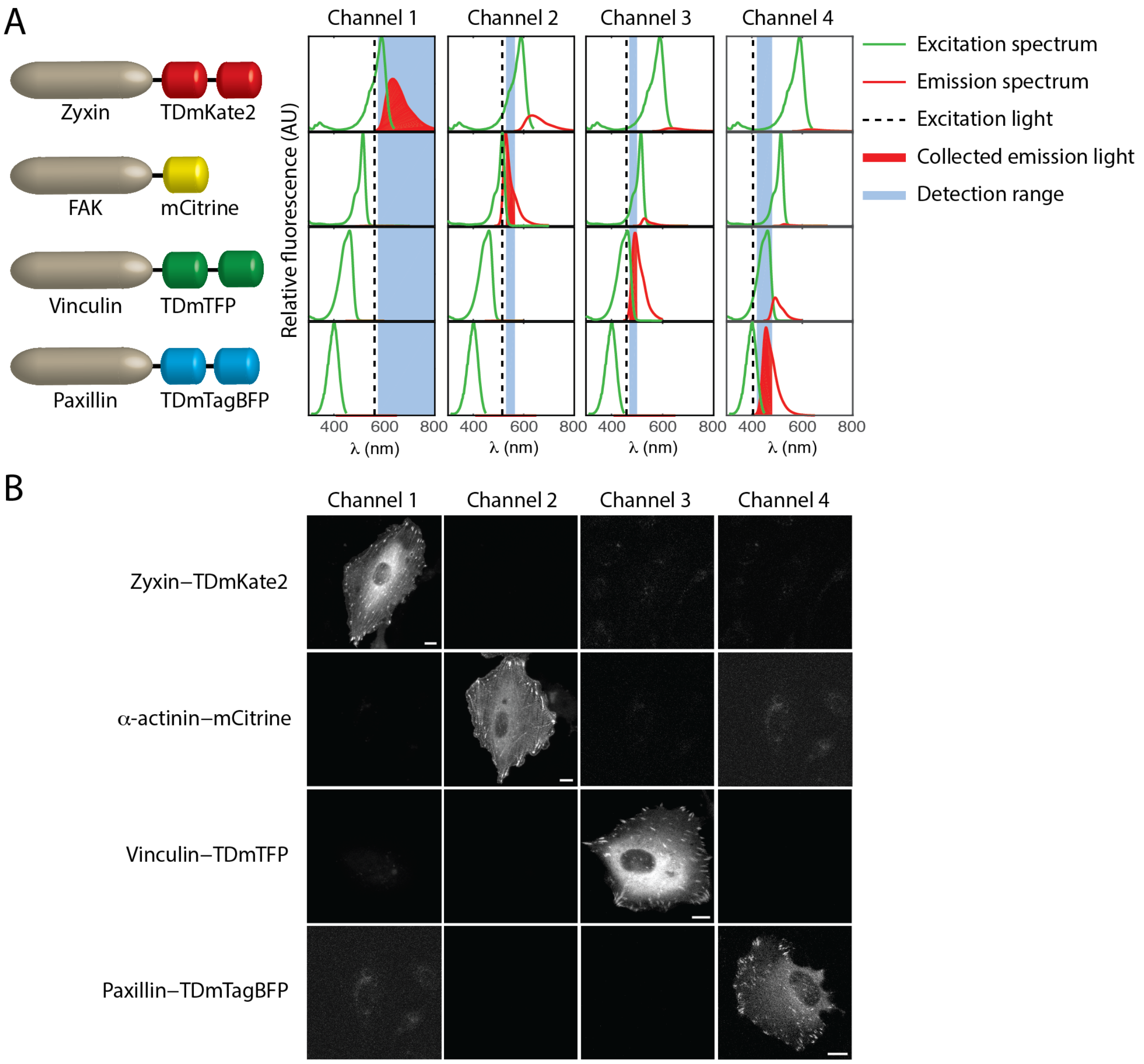

Figure S1. (A) The labeling scheme and the optical setup for the four-color live cell imaging. (B) Bleed-through matrix of the four-color imaging setup. REF52 cells were transfected with one type of plasmid as indicated and subjected to the four-color image acquisition. Scale bars, 10 μm.

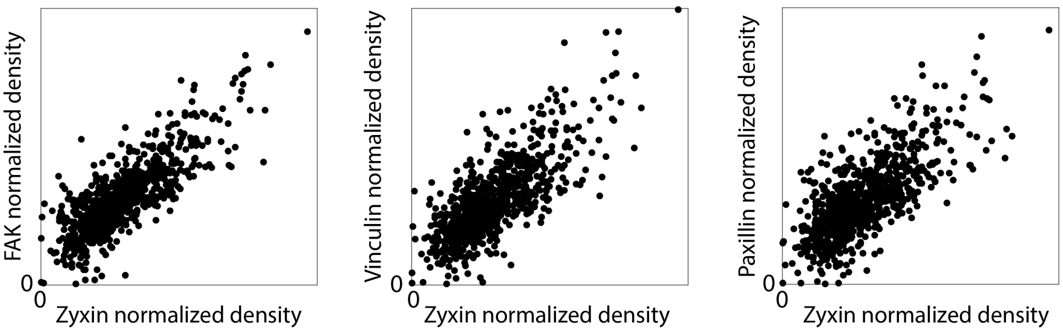

Figure S2. The relative levels of zyxin in the analyzed adhesion sites. Scatter plots showing the normalized densities of zyxin versus the normalized densities of FAK, vinculin or paxillin in the analyzed adhesion sites of the ten imaged cells at the first time point of the four-color time-lapse imaging ($n = 816$ focal adhesions).

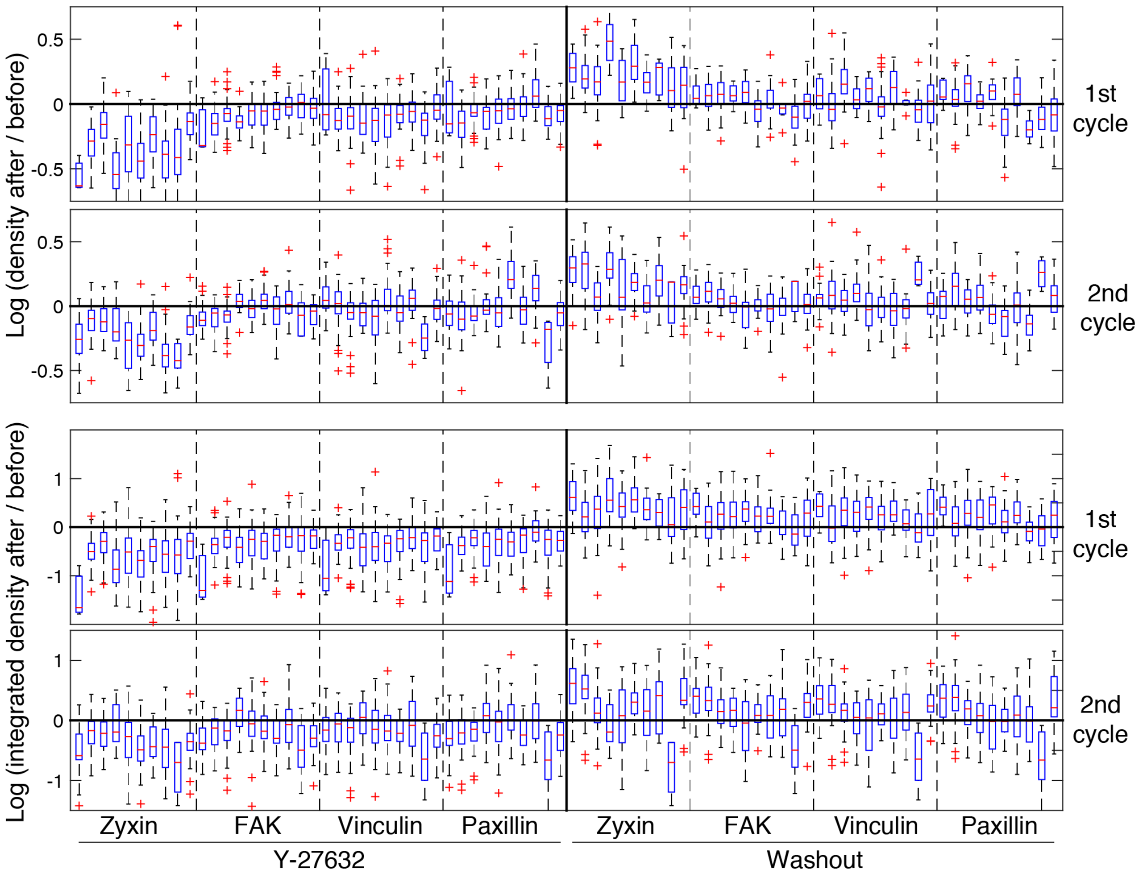

Figure S3. Box plots of the changes in proteins density and integrated density upon each perturbation, for the total population of focal adhesions in each of the ten analyzed cell (ten box plots within each dashed-line partition). The "before" and "after" density (or integrated density) values were averaged along 3 sequential time frames before the perturbation and at 24 minutes after the perturbation, respectively. In all box plots, boxes indicate the interquartile range (IQR) between the first and third quartiles and the line within indicates the median. Whiskers denote the lowest and highest values within 1.5*IQR from the first and third quartiles, respectively. Crosses indicate data points beyond the whiskers.

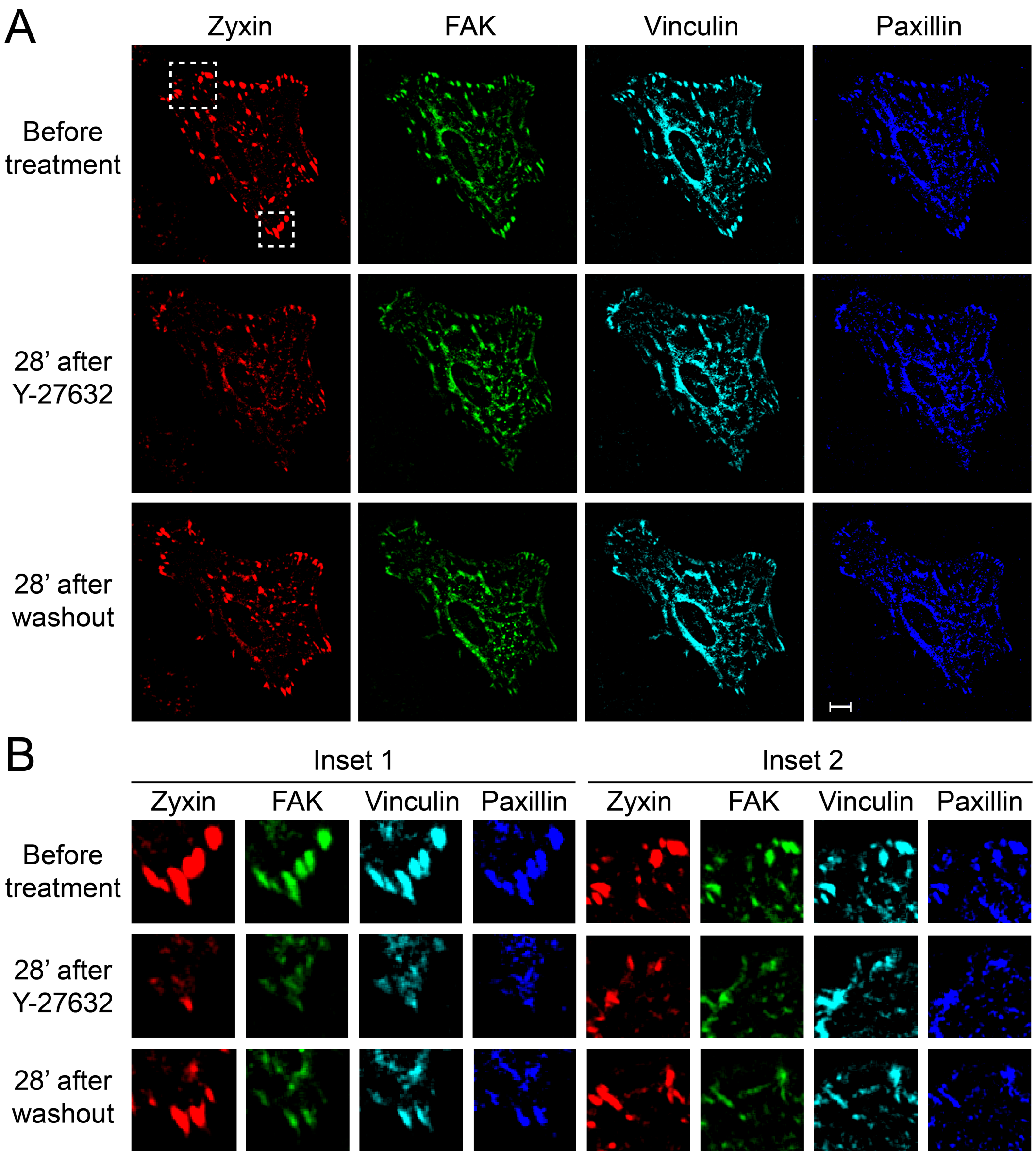

Figure S4. Four-color images showing the localizations of zyxin, FAK, vinculin and paxillin in the cell presented in Figure 3C at the corresponding time points. (A) Four-color images of the whole cell. Scale bar, 10 μm. (B) Magnifications of the two regions that are indicated in (A) by dashed rectangles, illustrating the changes in focal adhesions upon ROCK perturbations.

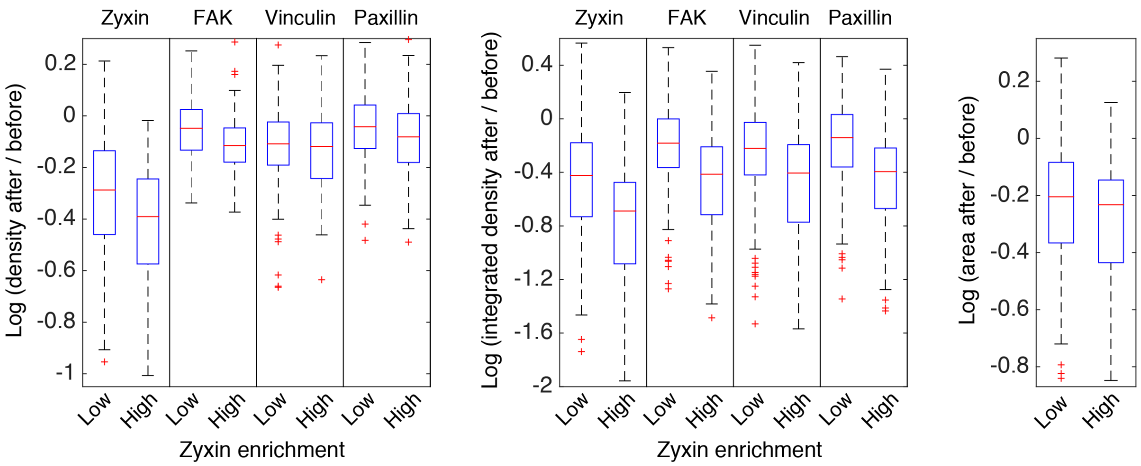

Figure S5. Comparisons between the responses of focal adhesions having low or high relative levels of zyxin. The traces of zyxin, FAK, vinculin and paxillin densities in single focal adhesions of the ten analyzed cells were normalized by dividing each trace by its mean. Focal adhesions were classified as having high or low zyxin enrichment if the normalized density of zyxin before the first perturbation was higher, or lower, than the normalized densities of FAK, vinculin and paxillin, respectively. Box plots show the log fold change in the density (left) and integrated density (middle) of zyxin, FAK, vinculin and paxillin, as well as in the area (right) of the focal adhesions, upon the first addition of Y-27632. The "before" and "after" values were averaged along 3 sequential time frames before the perturbation and at 24 minutes after Y-27632 addition, respectively. In all box plots, boxes indicate the interquartile range (IQR) between the first and third quartiles and the line within indicates the median. Whiskers denote the lowest and highest values within 1.5*IQR from the first and third quartiles, respectively. Crosses indicate data points beyond the whiskers.

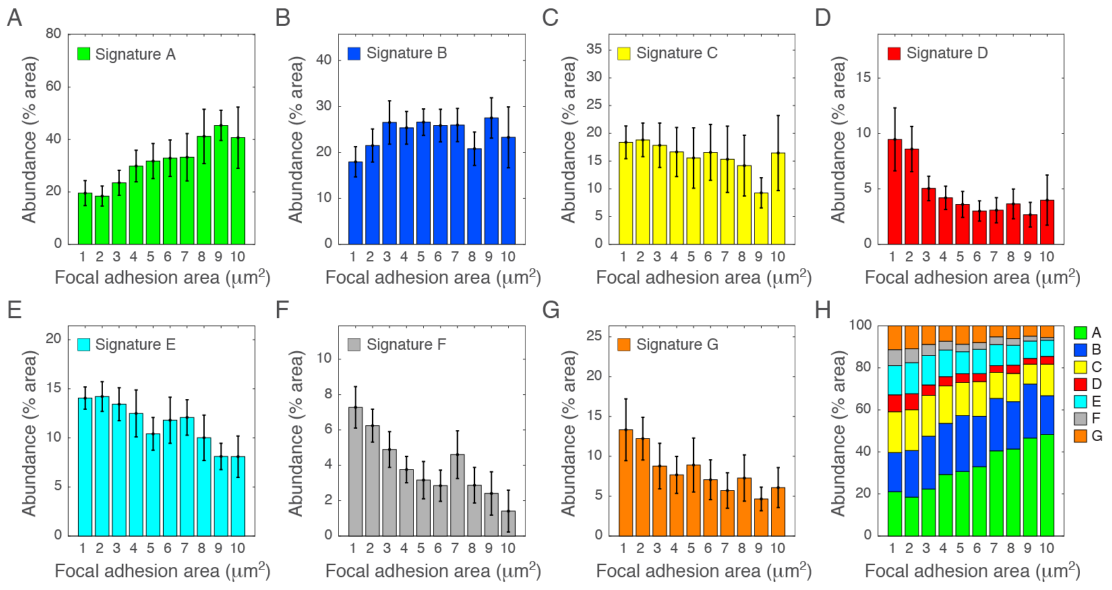

Figure S6. Correlations between the size of focal adhesions and the abundance of compositions within them. Focal adhesions from 5 cells were grouped into 10 area categories, ranging from 1 μm^2 to 10 μm^2. (A) - (G) Bar plots showing for each composition signature its relative abundance within each focal-adhesion area category. This relative abundance was calculated as the percentage of the area within a focal adhesion containing this composition signature out of the total area of the focal adhesion. Error bars indicate standard error of the mean (n = 5 cells). Color codes and composition signature names are consistent with Figure 3B. (H) A stacked bar plot of the relative abundance of the composition signatures in each focal adhesion area category.

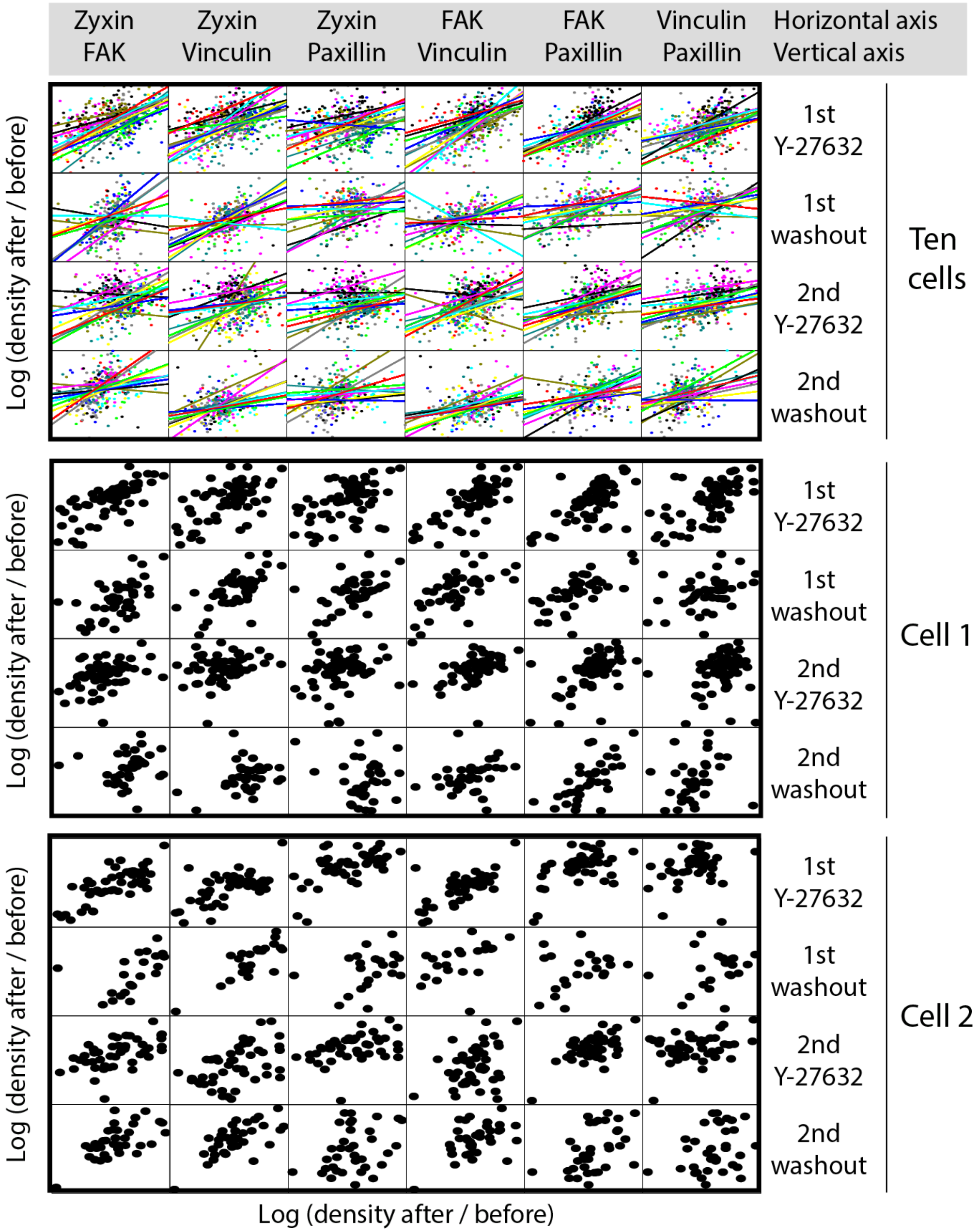

Figure S7. Correlations between the response extents of zyxin, FAK, vinculin and paxillin to ROCK perturbations. Response extents were calculated per focal adhesion and protein as log (density 14' after perturbation / density before perturbation). For robust quantification of responses per individual focal adhesions, the values of 3 sequential time frames were averaged before and 14 minutes after each perturbation. The scatter plots show the response extents of two proteins versus each other (as indicated at the top) of focal adhesions from ten cells or from single cells upon each perturbation (as indicated on the right). Overlaid at the top panel are linear regression lines fitted to data of each cell and color-coded accordingly.

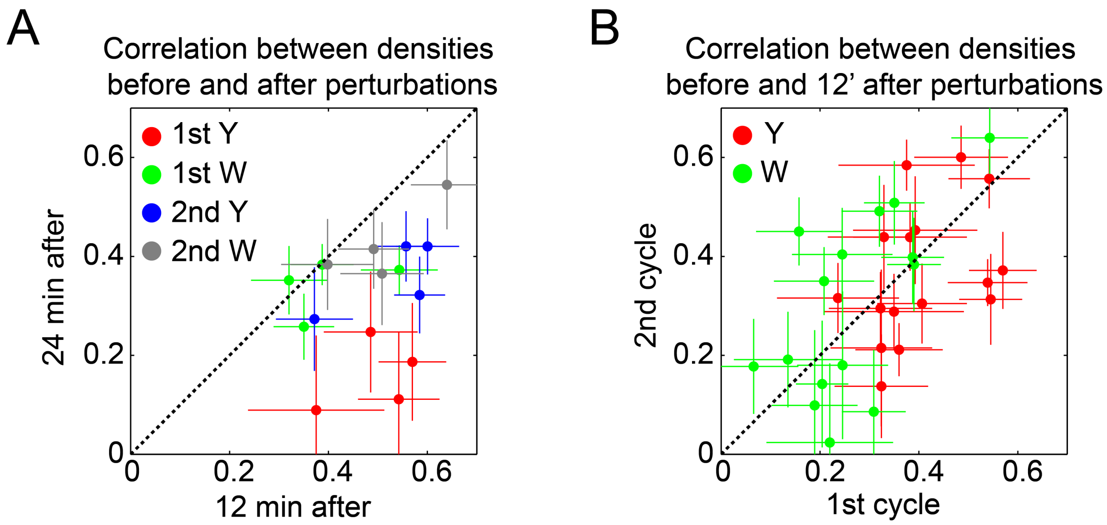

Figure S8. Scatter plots comparing the correlations of the pre- and post- perturbation densities, between different categories as indicated. Error bars indicate standard error of the mean (n = 10 cells). (A) Pearson correlation coefficients between the pre- and post- perturbation densities of the same protein. (B) Pearson correlation coefficients between the pre- and post- perturbation densities of the same proteins as well as of between different proteins. In all plots, before and after densities were averaged along 3 sequential time frames before and at the indicated time point after each perturbation, respectively.

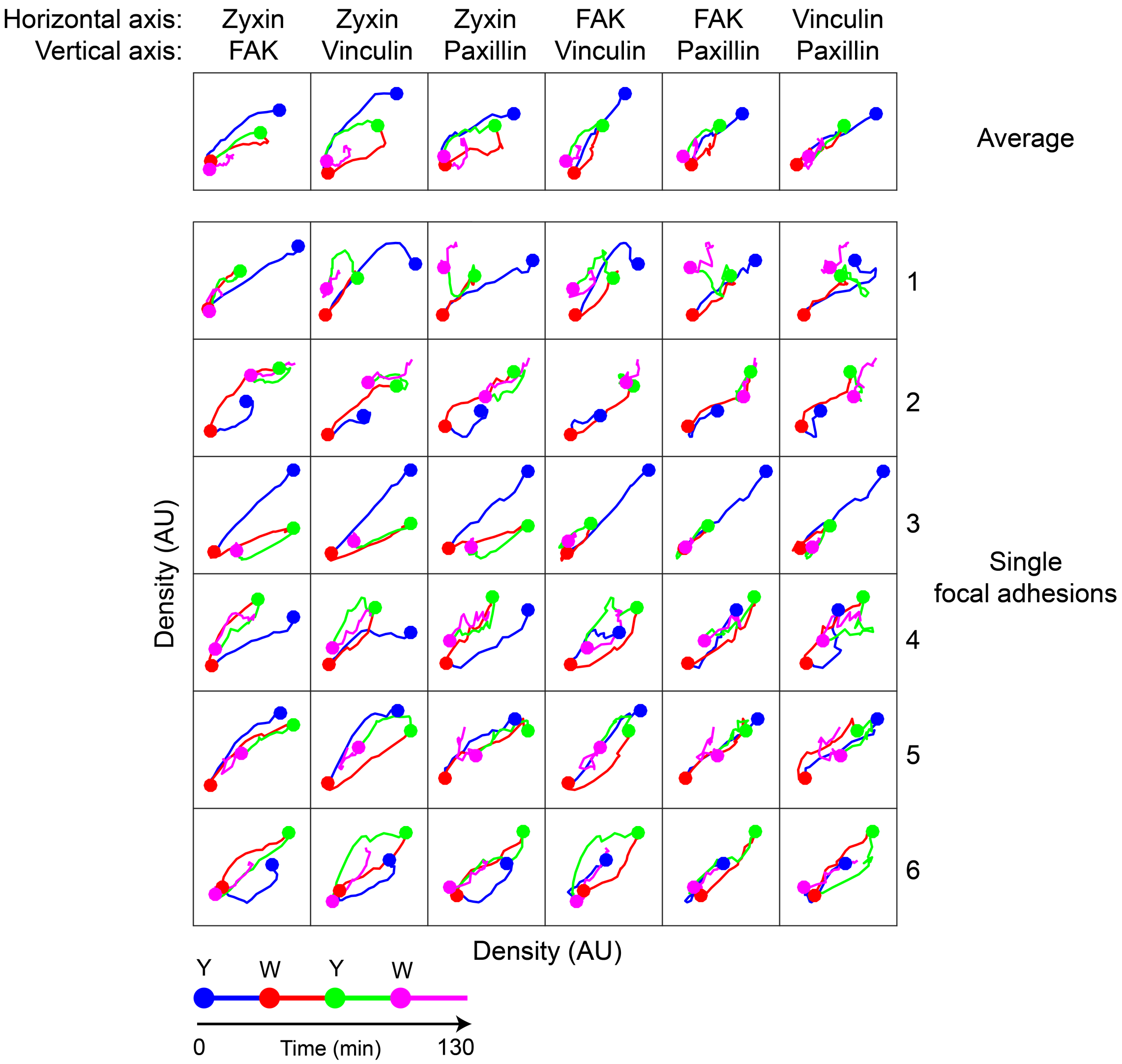

Figure S9. Phase-space plots comparing pairwise the densities of zyxin, FAK, vinculin and paxillin, averaged over a population of focal adhesions (top row, n = 58 focal adhesions) or for the six single focal adhesions of the same cell that were shown in Fig. 5B (bottom panel). The time-points of Y-27632 (Y) additions and washouts (W) are indicated color-coded by filled circles.

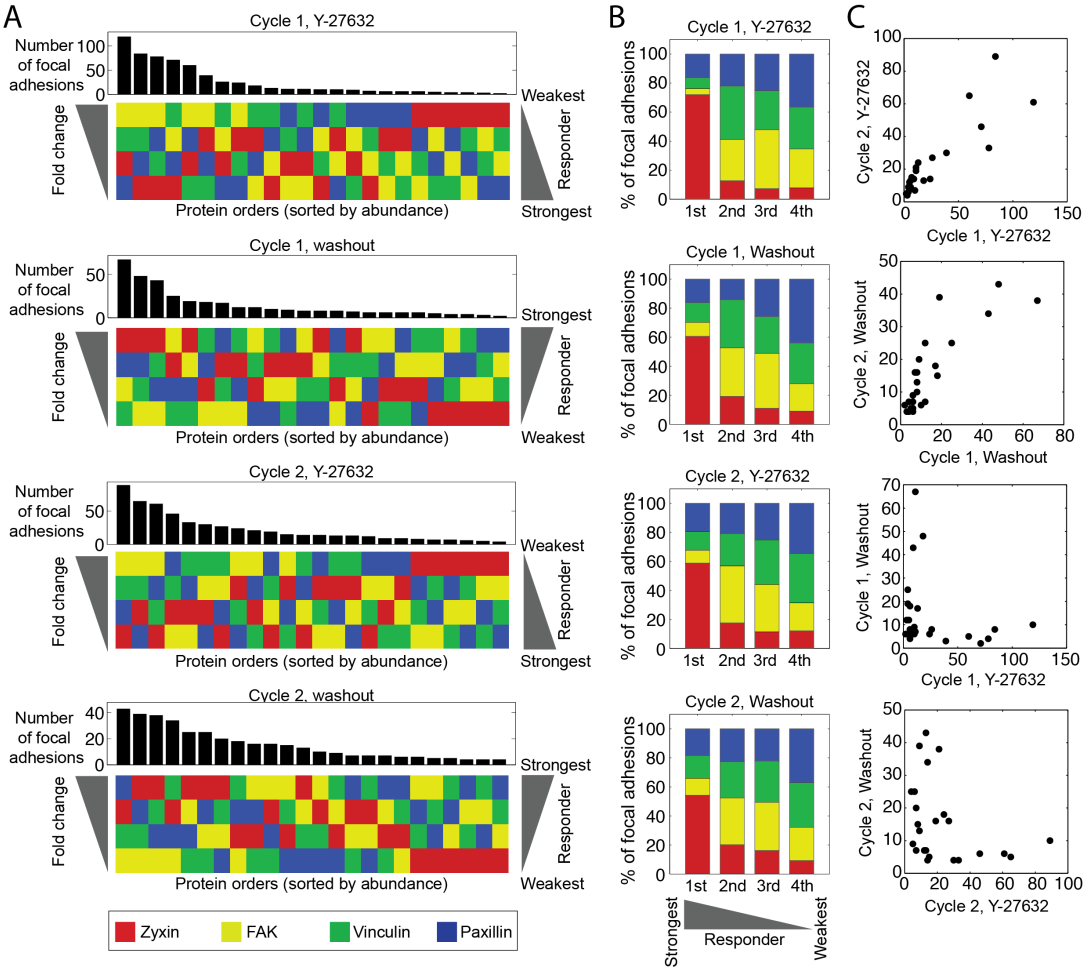

Figure S10. (A) Bar plots showing for each perturbation the total number of focal adhesions, from 10 cells, exhibiting a given hierarchy of proteins fold change ranks, as indicated by the heatmaps below. Since fold change is calculated as log(density after/before perturbation), a higher fold change implies a weaker response in the case of Y-27632 addition and a stronger response in the case of washout, as indicated on the right. (B) Stacked bar plots showing the percentages of focal adhesions having a given protein at a given place in the hierarchy of response strengths. Proteins are color-coded as in (A). (C) Scatter plots comparing the abundance of each fold change rank order between the indicated pairs of perturbations. For robust quantification of responses per individual focal adhesions, the values of 3 sequential time frames were averaged before perturbation and at 14 minutes after each perturbation.
